# Stochastic approximations of higher-molecular by bi-molecular reactions

**DOI:** 10.1007/s00285-022-01848-7

**Published:** 2023-01-13

**Authors:** Tomislav Plesa

**Affiliations:** Department of Applied Mathematics and Theoretical Physics, Centre for Mathematical Sciences, University of Cambridge, Wilberforce Road, Cambridge, CB3 0WA UK

**Keywords:** Stochastic reaction networks, Chemical master equation, Singular perturbation theory, Synthetic biology, DNA computing, 92B05, 92C42, 60J28

## Abstract

Reactions involving three or more reactants, called higher-molecular reactions, play an important role in mathematical modelling in systems and synthetic biology. In particular, such reactions underpin a variety of important bio-dynamical phenomena, such as multi-stability/multi-modality, oscillations, bifurcations, and noise-induced effects. However, as opposed to reactions involving at most two reactants, called bi-molecular reactions, higher-molecular reactions are biochemically improbable. To bridge the gap, in this paper we put forward an algorithm for systematically approximating arbitrary higher-molecular reactions with bi-molecular ones, while preserving the underlying stochastic dynamics. Properties of the algorithm and convergence are established via singular perturbation theory. The algorithm is applied to a variety of higher-molecular biochemical networks, and is shown to play an important role in synthetic biology.

## Introduction

Reaction networks (Feinberg 1979; Érdi and Tóth 1989) are a central mathematical framework for analyzing biochemical processes in systems biology (Vilar et al. 2002; Dublanche et al. 2006; Kar et al. 2009), and are a powerful programming language for designing molecular systems in synthetic biology (Soloveichik et al. 2010, 2008; Plesa et al. 2021, 2016, 2018; Srinivas et al. 2017). Not all reactions are equally likely to occur in biochemistry; in particular, reactive collisions between three or more molecules (higher-order/higher-molecular reactions) are less likely to take place than reactive collisions between two molecules (second-order/bi-molecular reactions) (Gillespie 1992). This fact is reflected, for example, in nucleic-acid-based synthetic biology—a branch of synthetic biology which utilizes nucleic acids (DNA or RNA molecules) to experimentally implement abstract reaction networks (Zhang and Winfree 2009)—where only up to second-order reactions have been rigorously shown to be experimentally realizable (Soloveichik et al. 2010). Despite being less biochemically plausible, higher-order reactions have nevertheless been used to mathematically model a variety of processes in both systems and synthetic biology. For example, third-order (tri-molecular) reactions appear in the one-species Schlögl system (Schlögl 1972), where they allow for bi-stability (coexistence of two stable equilibria), in the Brusselator (Prigogine and Lefever 1968) and Schnakenberg (Schnakenberg 1979) systems, which display oscillations (existence of a stable limit cycle), as well as in two-species biochemical networks displaying bicyclicity (coexistence of two stable limit cycles) (Plesa et al. 2017), and homoclinic (Plesa et al. 2016) and SNIC (Erban et al. 2009) bifurcations. Aside from well-mixed settings, third-order reactions also play a role in pattern formation (Cao and Erban 2014) and, more broadly, are a subject of research within reaction-diffusion bio-modelling (Li et al. 2018). In context of synthetic biology, higher-order reactions appear in the noise-control algorithm (Plesa et al. 2018) and the stochastic morpher controller (Plesa et al. 2021), where they respectively allow for local and global reshaping of the probability distributions for the abundance of molecular species.

An algorithm for approximating higher-order reactions with second-order ones at the *deterministic level*, i.e. at the level of the reaction-rate equations (Feinberg 1979), has been used for decades (Tyson 1973; Cook et al. 1989; Wilhelm 2000). This algorithm relies on suitable time-scale separations, and has been formally justified for third- and fourth-order reactions using perturbation theory (Cook et al. 1989; Wilhelm 2000). An alternative order-reduction algorithm has been presented in Kerner (1981), Kowalski (1993) which, instead of relying on time-scale separations, relies on appropriate initial conditions for some of the underlying species. Let us note that, from the perspective of synthetic biology, such sensitivity to initial conditions may pose significant experimental challenges (Weitz et al. 2014; Genot et al. 2016). Less attention has been paid to validity of such approximations at the *stochastic level*, i.e. at the level of the chemical master equation (CME) (Gillespie 1992). In this context, it has been formally shown in Janssen (1989) that a specific third-order reaction, namely $3 X \rightarrow 2 X$, can be stochastically approximated with a second-order network by applying the algorithm from Tyson (1973), Cook et al. (1989), and Wilhelm (2000); this result has also been qualitatively described in Gillespie (1992). However, the questions of convergence and whether the formal deterministic results from Tyson (1973), Cook et al. (1989), and Wilhelm (2000) extend into the stochastic regime for arbitrary reactions remain unanswered. In particular, validity of perturbation results at the deterministic level does not necessarily imply validity at the stochastic level (Thomas et al. 2011; Kim et al. 2014; Agarwal et al. 2012). To bridge the gap, in this paper we establish properties, including convergence estimates, for the algorithm from Tyson (1973), Cook et al. (1989), and  Wilhelm (2000) in context of reactions of arbitrary order at the stochastic level.

The paper is organized as follows. In Sect. 2, we prove that any one-species third-order reaction can be approximated with a suitable family of second-order networks, and we apply the results in Sect. 3 on the Schlögl system (Schlögl 1972). In Sect. 4, we generalize the results from Sect. 2 to arbitrary multi-species higher-order reactions under mass-action kinetics. In Sect. 5, we apply the generalized results to higher-order reaction networks displaying noise-induced phenomena. Finally, we conclude with a summary and discussion in Sect. 6. The notation and background theory used in the paper are introduced as needed, and are summarized in Appendix 1. More detailed analyses underlying Sect. 4 are provided in Appendices 2–3.

## Special case: one-species third-order reactions

Let us consider an arbitrary *input* reaction network $\mathcal {R}_0 = \mathcal {R}_0(X)$, under mass-action kinetics (Feinberg 1979), involving a single biochemical species *X*, given by1where $\nu _j \in {\mathbb {Z}}_{\ge }$ and $\bar{\nu }_j \in {\mathbb {Z}}_{\ge }$ are the reactant and product stoichiometric coefficients of the *j*-reaction, respectively, while $k_j \in \mathbb {R}_{>}$ is the corresponding dimensionless rate coefficient; ${\mathbb {Z}}_{\ge }$ and $\mathbb {R}_{>}$ are the sets of nonnegative integers and positive real numbers, respectively. See also Appendix 1 for notation and reaction network theory. Here,  is an arbitrary one-species third-order (tri-molecular) reaction, which we wish to approximate, while $\mathcal {R}_{\rho } = \mathcal {R}_{\rho }(X)$, called the residual network, contains the remaining reactions from $\mathcal {R}_0$ that we do not wish to approximate. Let us now consider the *output* mass-action network $\mathcal {R}_{\varepsilon } = \mathcal {R}_{\varepsilon }(X, Y)$, containing an auxiliary species *Y*, given by2where we denote the irreversible forward and backward reactions  and , respectively, jointly as a single reversible reaction . In particular, the output network $\mathcal {R}_{\varepsilon }$ is obtained from the input network $\mathcal {R}_0$ by replacing the target tri-molecular reaction  with the second-order (bi-molecular) sub-network $\mathcal {R}_{1}^{\varepsilon } \cup \mathcal {R}_{2}$, while leaving the residual network $\mathcal {R}_{\rho }$ unchanged. Let us note that the network $\mathcal {R}_{1}^{\varepsilon } \cup \mathcal {R}_{2}$ is said to be of second-order because its highest-order reaction is of second-order. Formally speaking, when the backward reaction from $\mathcal {R}_{1}^{\varepsilon }$ is sufficiently fast (i.e. the parameter $\varepsilon > 0$ is sufficiently small), then the short-lived auxiliary species *Y* encodes the complex 2*X*. Consequently, the left-hand side of the output reaction $\mathcal {R}_{2}$ then formally becomes 3*X*, thus mimicking the target input reaction; see also Example 4.1 in Sect. 4, where this formal approach is discussed more generally for higher-molecular networks. More precisely, in what follows we prove that, under suitable conditions on the kinetic and stoichiometric coefficients $\kappa _1, \kappa _2 \in \mathbb {R}_{>}$ and $\tilde{\nu }, \tilde{\gamma } \in {\mathbb {Z}}_{\ge }$, respectively, the *x*-marginal probability-mass function (PMF) of the output network (2) approaches the PMF of the input network (1) as $\varepsilon \rightarrow 0$, which we formulate as Corollary 2.1 in Sect. 2.2.

### Perturbation analysis

Let us denote the copy-numbers of species $\{X, Y\}$ by $(x, y)^{\top } \in {\mathbb {Z}}_{\ge }^{2}$, and the time-variable by $t \in \mathbb {R}_{\ge }$. Under suitable conditions, the PMF of reaction network (2), denoted by $p_{\varepsilon }(x,y,t)$, satisfies a partial difference-differential equation called the *chemical master equation* (CME) (Gillespie 1992; Erban et al. 2019; Van Kampen 2007), see also Appendix 1. As motivated shortly, we introduce new coordinates $\bar{x} = (x + 2 y)$ and $\tau = \varepsilon t$, in which the CME for (2) reads

3 $$\begin{aligned} \frac{\textrm{d}}{\textrm{d} \tau } p_{\varepsilon }(\bar{x},y,\tau )&= \left( \frac{1}{\varepsilon ^{2}}\mathcal {L}_0 + \frac{1}{\varepsilon } (\mathcal {L}_1 + \mathcal {L}_2) + \bar{\mathcal {L}}_{\rho } \right) p_{\varepsilon }(\bar{x},y,\tau ), \; \; \text {where } \bar{x} = x + 2 y. \end{aligned}$$

Here, operator $\mathcal {L}_0$ is induced by the backward reaction from $\mathcal {R}_{1}^1$, $\mathcal {L}_1$ by the forward reaction from $\mathcal {R}_{1}^1$, $\mathcal {L}_2$ by $\mathcal {R}_2$, and $\bar{\mathcal {L}}_{\rho }$ is induced by the residual network $\mathcal {R}_{\rho }$ with suitably rescaled rate coefficients:

4 $$\begin{aligned} \mathcal {L}_0&= \left( E_{y}^{+1} - 1 \right) y, \nonumber \\ \mathcal {L}_{1}&= \left( E_{y}^{-1} - 1 \right) \alpha _1(\bar{x},y), \; \; \text {where } \alpha _1(\bar{x},y) = \kappa _1 (\bar{x} - 2 y)^{\underline{2}}, \nonumber \\ \mathcal {L}_{2}&= \left( E_{\bar{x}}^{-(\tilde{\nu } + 2 \tilde{\gamma } - 3)} E_{y}^{- (\tilde{\gamma } - 1)} - 1 \right) \alpha _{2}(\bar{x},y) y, \; \; \text {where } \alpha _2(\bar{x},y) = \kappa _2 (\bar{x} - 2 y), \nonumber \\ \bar{\mathcal {L}}_{\rho }&= \sum _{j = 2}^M \left( E_{\bar{x}}^{- (\bar{\nu }_j - \nu _j)} - 1 \right) \varepsilon ^{-1} \beta _j(\bar{x}, y), \; \; \text { where } \varepsilon ^{-1} \beta _j(\bar{x}, y) = (\varepsilon ^{-1} k_j) (\bar{x} - 2 y)^{\underline{\nu _j}}, \end{aligned}$$

where $x^{\underline{\nu }} = x (x - 1) \ldots (x - \nu + 1)$, and where the step operator $E_{x}^{\Delta x}$ is such that $E_{x}^{\Delta x} f(x) = f(x + \Delta x)$ for any sequence $f : {\mathbb {Z}} \rightarrow \mathbb {R}$.

We now perform a formal perturbation analysis (Pavliotis and Stuart 2008) of the CME (3) in the limit $\varepsilon \rightarrow 0$, which we rigorously justify in the next section. To this end, let us consider the perturbation series

5 $$\begin{aligned} p_{\varepsilon }(\bar{x},y,\tau )&= p_0(\bar{x},y,\tau ) + \varepsilon p_1(\bar{x},y,\tau ) + \varepsilon ^2 p_2(\bar{x},y,\tau ) + \ldots , \end{aligned}$$

where we require that the zero-order term is a PMF, i.e. $p_0(\cdot , \cdot ,\tau ) : {\mathbb {Z}}_{\ge }^{2} \rightarrow [0,1]$ and $\langle 1, p_0(\cdot ,\cdot ,\tau ) \rangle = 1$ for all $\tau \ge 0$, where $\langle f, g\rangle \equiv \sum _{\textbf{x} \in {\mathbb {Z}}_{\ge }^N} f(\textbf{x}) g(\textbf{x})$ for any two sequences $f, g: {\mathbb {Z}}_{\ge }^N \rightarrow \mathbb {R}$. Substituting (5) into (3) and equating terms of equal powers in $\varepsilon$, one obtains the following system of equations:

6 $$\begin{aligned} \mathcal {O} \left( \varepsilon ^{-2}\right) : \; \mathcal {L}_{0} p_0(\bar{x},y,\tau )&= 0, \end{aligned}$$

7 $$\begin{aligned} \mathcal {O} \left( \varepsilon ^{-1}\right) : \; \mathcal {L}_{0} p_{1}(\bar{x},y,\tau )&=- (\mathcal {L}_1 + \mathcal {L}_2) p_{0}(\bar{x},y,\tau ), \end{aligned}$$

8 $$\begin{aligned} \mathcal {O} \left( 1\right) : \; \mathcal {L}_{0} p_{2}(\bar{x},y,\tau )&= \left( \frac{\textrm{d}}{\textrm{d} \tau } - \bar{\mathcal {L}}_{\rho }\right) p_{0}(\bar{x},y,\tau ) - (\mathcal {L}_1 + \mathcal {L}_2) p_{1}(\bar{x},y,\tau ). \end{aligned}$$

Equation (6). Since operator $\mathcal {L}_0$ acts and depends only on *y*, we seek the zero-order term in a separable form, $p_0(\bar{x},y,\tau ) = p_0(\bar{x},\tau ) p_0(y)$, which gives

9 $$\begin{aligned} p_0(\bar{x},y,\tau )&= p_0(\bar{x},\tau ) \delta _{y, 0}, \end{aligned}$$

where $\delta _{y,0}$ is the Kronecker-delta function centered at zero, i.e. $\delta _{y,0} = 1$ if $y = 0$, and $\delta _{y,0} = 0$ otherwise; see also Appendix 1.

*Remark*. In the original coordinates (*x*, *y*), operator $\mathcal {L}_0$ corresponds to the reaction , and equation (6) has infinitely many solutions. This degeneracy arises from the fact that the process induced by $\mathcal {L}_0$ satisfies a local linear conservation law $x + 2 y = \bar{x}$, where $\bar{x}$ is time-independent. Using this conservation law as a coordinate change leads to equation (6), where $\bar{x}$ is only a parameter, with the solution $p_0(y) = \delta _{y,0}$ which is unique up to a multiplicative $(\bar{x}, \tau )$-dependent constant.

*Remark*. Let $l_0^K = \{p : {\mathbb {Z}}_{\ge } \rightarrow \mathbb {R} \, | \, p(y) = 0 \; \; \forall y \ge K\}$ be the space of sequences whose elements beyond $K \ge 0$ are zero. If $\mathcal {L}_0$ is as given in (4) and if $f \in l_0^K$, then any solution of $\mathcal {L}_0 p = f$ satisfies $p \in l_0^K$. Therefore, since $p_0(\bar{x},\cdot ,\tau ) \in l_0^1$, it follows that (7) and (8) are both finite-dimensional systems of linear equations with respect to the variable *y* for any parameter choice $(\bar{x}, \tau )$.

Equation (7). Using (4) and (9), it follows that

10 $$\begin{aligned} (\mathcal {L}_1 + \mathcal {L}_2) p_0(\bar{x},y,\tau )&= \mathcal {L}_1 p_0(\bar{x},y,\tau ) = p_0(\bar{x},\tau ) \left( - \alpha (\bar{x}, 0) \delta _{y,0} + \alpha (\bar{x}, 0) \delta _{y,1}\right) . \end{aligned}$$

Considering the form of $\mathcal {L}_0$ and (10), we seek a solution of (7) in a separable form, $p_1(\bar{x},y,\tau ) = p_0(\bar{x},\tau ) \left( c(\bar{x}) \delta _{y,0} + p_1(\bar{x}) \delta _{y,1} \right)$, where $c, p_1 : {\mathbb {Z}}_{\ge } \rightarrow \mathbb {R}$ are arbitrary. Substitution into (7) leads to the general solution

11 $$\begin{aligned} p_1(\bar{x},y,\tau )&= p_0(\bar{x},\tau ) \left( c(\bar{x}) \delta _{y,0} + \alpha _1(\bar{x},0) \delta _{y,1} \right) . \end{aligned}$$

Equation (8). As remarked previously, (8) is a finite-dimensional system of linear equations; in particular, $p_2(\bar{x},\cdot ,\tau ) \in l_0^{\text {max}\{2, \tilde{\gamma }\}+1}$; therefore, the Fredholm alternative theorem holds (Kreyszig 1989). The adjoint (backward) operator corresponding to $\mathcal {L}_0$ is given by $\mathcal {L}_0^* = y \left( E_{y}^{-1} - 1 \right)$, and its null-space $\mathcal {N}$ is given by $\mathcal {N}(\mathcal {L}_0^*) = \{1\}$; see also Appendix 1. Therefore, it follows from the Fredholm alternative theorem that (8) has a solution if and only if the solvability condition $\langle 1, \text {RHS} \rangle _y = 0$ holds, where $\langle 1, \cdot \rangle _y = \sum _{y \in {\mathbb {Z}}_{\ge }} \cdot$, and $\text {RHS}$ denotes the right-hand side of (8); substituting (4), (9), and (11) into the solvability condition, one obtains the *effective* CME

12 $$\begin{aligned} \frac{\textrm{d}}{\textrm{d} t} p_0(\bar{x},t)&= \left[ \left( E_{\bar{x}}^{-(\tilde{\nu } + 2 \tilde{\gamma } - 3)} - 1 \right) \varepsilon \kappa _1 \kappa _2 \bar{x} (\bar{x} - 1) (\bar{x} - 2) +\mathcal {L}_{\rho } \right] p_0(\bar{x},t), \end{aligned}$$

where $\mathcal {L}_{\rho }$ is the forward operator induced by the residual network $\mathcal {R}_{\rho }$.

*Remark*. In the original coordinate *t*, the Fredholm alternative theorem applied to (7) enforces a trivial effective CME $\textrm{d}/\textrm{d} t \, p_0(\bar{x},t) = 0$; to capture non-trivial dynamics, we have rescaled time to a longer scale.

*Remark*. Equation (12) describes a time-evolution of the PMF for the stochastic process $\bar{X}(t) = X(t) + 2 Y(t)$, and not the original copy-number *X*(*t*). However, (9) implies that process *Y*(*t*) spends most of the time at $y = 0$ as $\varepsilon \rightarrow 0$, so that the PMFs for $\bar{X}(t)$ and *X*(*t*) match as $\varepsilon \rightarrow 0$.

### Kinetic and stoichiometric conditions

To ensure that the dynamics of the output network (2) matches that of the input network (1), coefficients $\kappa _1$ and $\kappa _2$ have to suitably scale with $\varepsilon$. In particular, the CME for (1) reads

13 $$\begin{aligned} \frac{\textrm{d}}{\textrm{d} t} p(x,t)&= \left[ \left( E_{x}^{-(\bar{\nu }_1 - 3)} - 1 \right) k_1 x (x - 1) (x - 2) + \mathcal {L}_{\rho } \right] p(x,t). \end{aligned}$$

In order for (12) and (13) to match, we impose the *kinetic condition*, given by

14 $$\begin{aligned} \varepsilon \kappa _1 \kappa _2&= k_1, \; \; \text {where } \kappa _1, \kappa _2 = o(\varepsilon ^{-1}) \; \; \text {as } \varepsilon \rightarrow 0, \end{aligned}$$

and the *stoichiometric condition*, given by

15 $$\begin{aligned} \tilde{\nu }&= \bar{\nu }_1 - 2 \tilde{\gamma }, \end{aligned}$$

where $o(\cdot )$ is the “little-o" asymptotic symbol, see also Appendix 1.

*Remark*. Requirements $\kappa _1 = o(\varepsilon ^{-1})$ and $\kappa _2 = o(\varepsilon ^{-1})$ as $\varepsilon \rightarrow 0$ from (14) respectively ensure that the operators $\mathcal {L}_1$ and $\mathcal {L}_2$ remain slower than $\varepsilon ^{-1} \mathcal {L}_0$ in (3); otherwise, in the degenerate case when $\kappa _1 = \mathcal {O}(\varepsilon ^{-1})$ or $\kappa _2 = \mathcal {O}(\varepsilon ^{-1})$, where $O(\cdot )$ is the “big-O" symbol, one obtains families of perturbation problems distinct from the one considered in this section.

### Convergence

The formal perturbation analysis from Sect. 2.1 has been performed under the assumption that $t = \mathcal {O}(\varepsilon ^{-1})$, $\{k_j = \mathcal {O}(\varepsilon )\}_{j = 2}^M$, and that $\kappa _1$ and $\kappa _2$ are independent of $\varepsilon$, which is inconsistent with the kinetic condition (14). We stress that an objective of the analysis in Sect. 2.1 was precisely to uncover admissible $\varepsilon$-scalings of $\kappa _1$ and $\kappa _2$ which ensure that (1) and (2) match. Having formally obtained such candidates, we now perform a convergence analysis, without the aforementioned assumptions, under a particular scaling which satisfies (14), given by

16 $$\begin{aligned} \kappa _1&= \bar{\kappa }_1 \varepsilon ^{-1/2}, \; \; \; \kappa _2 = \bar{\kappa }_2 \varepsilon ^{-1/2}, \end{aligned}$$

where $\bar{\kappa }_1, \bar{\kappa }_2$ are suitable $\varepsilon$-independent parameters. The CME for network (2) under (16) reads

17 $$\begin{aligned} \frac{\textrm{d}}{\textrm{d} t} p_{\varepsilon }(\bar{x},y,t)&= \mathcal {L}_{\varepsilon } p_{\varepsilon }(\bar{x},y,t) = \left( \frac{1}{\varepsilon }\mathcal {L}_0 + \frac{1}{\varepsilon ^{1/2}} (\mathcal {L}_1 + \mathcal {L}_2) + \mathcal {L}_{\rho } \right) p_{\varepsilon }(\bar{x},y,t), \; \; \text {where } \bar{x} = x + 2 y. \end{aligned}$$

Substituting into (17) the fractional-power perturbation series

18 $$\begin{aligned} p_{\varepsilon }(\bar{x},y,t)&= p_0(\bar{x},y,t) + \varepsilon ^{1/2} p_1(\bar{x},y,t) + \varepsilon p_2(\bar{x},y,t) + \ldots , \end{aligned}$$

one obtains

19 $$\begin{aligned} \mathcal {O} \left( \varepsilon ^{-1} \right) : \; \mathcal {L}_{0} p_0(\bar{x},y,t)&= 0, \nonumber \\ \mathcal {O} \left( \varepsilon ^{-1/2} \right) : \; \mathcal {L}_{0} p_{1}(\bar{x},y,t)&=- (\mathcal {L}_1 + \mathcal {L}_2) p_{0}(\bar{x},y,t), \nonumber \\ \mathcal {O} \left( 1 \right) : \; \mathcal {L}_{0} p_{2}(\bar{x},y,t)&= \left( \frac{\textrm{d}}{\textrm{d} t} - \mathcal {L}_{\rho } \right) p_{0}(\bar{x},y,t) - (\mathcal {L}_1 + \mathcal {L}_2) p_{1}(\bar{x},y,t). \end{aligned}$$

Since systems (6)–(8) and (19) have the same form, the same is true for their solutions. In particular, the zero-order PMF from (19) is given by

20 $$\begin{aligned} p_0(\bar{x},y,t)&= p_0(\bar{x},t) \delta _{y, 0}, \end{aligned}$$

where the factor $p_0(\bar{x},t)$ satisfies

21 $$\begin{aligned} \frac{\textrm{d}}{\textrm{d} t} p_0(\bar{x},t)&= \left[ \left( E_{\bar{x}}^{-(\tilde{\nu } + 2 \tilde{\gamma } - 3)} - 1 \right) \bar{\kappa }_1 \bar{\kappa }_2 \bar{x} (\bar{x} - 1) (\bar{x} - 2) + \mathcal {L}_{\rho } \right] p_0(\bar{x},t). \end{aligned}$$

In what follows, we establish a weak convergence result over bounded domains; to this end, we let $\Vert p \Vert _{l_1(\mathbb {S}_{x} \times \mathbb {S}_{y})} \equiv \sum _{x \in \mathbb {S}_x} \sum _{y \in \mathbb {S}_y} |p(x, y) |$ denote the $l_1$-norm over a set $\mathbb {S}_{x} \times \mathbb {S}_{y} \subset {\mathbb {Z}}_{\ge }^{2}$. Furthermore, when convenient, we explicitly denote dependence of PMFs on the rate coefficients, e.g. $p_{\varepsilon } = p_{\varepsilon }(\bar{x},y,t; \, \varvec{\bar{\kappa }}, \textbf{k}_{\rho })$, where $\varvec{\bar{\kappa }} = (\bar{\kappa }_1, \bar{\kappa }_2)^{\top } \in \mathbb {R}_{>}^2$ and $\textbf{k}_{\rho } = (k_2, k_3, \ldots , k_M)^{\top } \in \mathbb {R}_{>}^{M-1}$.

#### Proposition 2.1

Consider the network $\mathcal {R}_{\varepsilon }$ (2) with rate coefficients $\kappa _1$ and $\kappa _2$ satisfying (16), whose PMF $p_{\varepsilon }(\bar{x},y,t; \, \varvec{\bar{\kappa }}, \textbf{k}_{\rho })$ satisfies (17). Let $p_0(\bar{x},y,t; \, \varvec{\bar{\kappa }}, \textbf{k}_{\rho })$ be the PMF satisfying (20)–(21). Assume that $p_{\varepsilon }(\bar{x},y,0; \, \varvec{\bar{\kappa }}, \textbf{k}_{\rho }) = p_0(\bar{x},y,0; \, \varvec{\bar{\kappa }}, \textbf{k}_{\rho })$. Then, for every compact parameter set ${\mathbb {K}} \subset \mathbb {R}_{>}^{M+1}$, compact state-space $\mathbb {S}_{\bar{x}} \times \mathbb {S}_{y} \subset {\mathbb {Z}}_{\ge }^{2}$, where $\mathbb {S}_y \supseteq [0,\text {max} \{2, \tilde{\gamma }\}]$, and compact time-interval [0, *T*], where $T> 0$, there exist constants $c > 0$ and $\varepsilon _0> 0$ such that for all $(\varvec{\bar{\kappa }}, \textbf{k}_{\rho })^{\top } \in {\mathbb {K}}$, $t \in [0,T]$ and $\varepsilon \in (0, \varepsilon _0]$

22 $$\begin{aligned} \left\| p_{\varepsilon }(\cdot ,\cdot ,t; \, \, \varvec{\bar{\kappa }}, \textbf{k}_{\rho }) - p_0(\cdot ,\cdot ,t; \, \varvec{\bar{\kappa }}, \textbf{k}_{\rho }) \right\| _{l_1(\mathbb {S}_{\bar{x}} \times \mathbb {S}_{y})}&\le c \, \varepsilon ^{1/2}. \end{aligned}$$

#### Proof

For every bounded set $\mathbb {S}_{\bar{x}} \times \mathbb {S}_{y} \subset {\mathbb {Z}}_{\ge }^{2}$, $p_{\varepsilon }(t) = p_{\varepsilon }(\cdot ,\cdot ,t)$ is a finite-dimensional vector. By assumption, $\mathbb {S}_y \supseteq [0,\text {max} \{2, \tilde{\gamma }\}]$; hence, the derivation from Sect. 2.1 can be reversed, i.e. there exist finite-dimensional vectors $p_1(t) = p_1(\cdot ,\cdot ,t)$ and $p_2(t) = p_2(\cdot ,\cdot ,t)$ such that (19) holds; in what follows, we also let $p_0(t) = p_0(\cdot ,\cdot ,t)$. Let us define a remainder $r_{\varepsilon }(t) = r_{\varepsilon }(\cdot , \cdot ,t)$ via

23 $$\begin{aligned} p_{\varepsilon }(t) = p_0(t) + \varepsilon ^{1/2} p_1(t) + \varepsilon p_2(t) + r_{\varepsilon }(t). \end{aligned}$$

Substituting (23) into (17), using (19) and the assumption that $p_0(0) = p_{\varepsilon }(0)$, one obtains an initial-value problem for the remainder:

24 $$\begin{aligned} \frac{\textrm{d}}{\textrm{d} t} r_{\varepsilon } (t) - \mathcal {L}_{\varepsilon } r_{\varepsilon } (t)&= \varepsilon ^{\frac{1}{2}} f_1(t) + \varepsilon f_2(t), r_{\varepsilon }(0) = -\left( \varepsilon ^{\frac{1}{2}} p_1(0) + \varepsilon p_2(0) \right) , \end{aligned}$$

where

25 $$\begin{aligned} f_1(t)&= \mathcal {L}_{\rho } p_1(t) -\frac{\textrm{d}}{\textrm{d} t} p_1(t) + (\mathcal {L}_1 + \mathcal {L}_2) p_2(t), \; \; \; f_2(t) = \mathcal {L}_{\rho } p_2(t) - \frac{\textrm{d}}{\textrm{d} t} p_2(t). \end{aligned}$$

Solving (24), applying $\Vert \cdot \Vert _{l_1(\mathbb {S}_{\bar{x}} \times \mathbb {S}_{y})}$ and the triangle inequality, and using the fact that $\Vert e^{\mathcal {L}_{\varepsilon } t} \Vert _{l_1(\mathbb {S}_{\bar{x}} \times \mathbb {S}_{y})} \le 1$, one obtains

26 $$\begin{aligned} \Vert r_{\varepsilon } (t) \Vert _{l_1(\mathbb {S}_{\bar{x}} \times \mathbb {S}_{y})}&\le \varepsilon ^{\frac{1}{2}} \left( \Vert p_1(0)\Vert _{l_1(\mathbb {S}_{\bar{x}} \times \mathbb {S}_{y})} + t \, \underset{0 \le s \le t}{\text {max}} \left\| f_1(s) \right\| _{l_1(\mathbb {S}_{\bar{x}} \times \mathbb {S}_{y})}\right) \nonumber \\&+ \varepsilon \left( \Vert p_2(0)\Vert _{l_1(\mathbb {S}_{\bar{x}} \times \mathbb {S}_{y})} + t \, \underset{0 \le s \le t}{\text {max}} \left\| f_2(s) \right\| _{l_1(\mathbb {S}_{\bar{x}} \times \mathbb {S}_{y})}\right) , \end{aligned}$$

where $\text {max}_{0 \le s \le t} g(s)$ denotes the maximum value of a continuous function *g*(*s*) for $s \in [0,t]$. The PMF $p_0(t; \, \varvec{\bar{\kappa }}, \textbf{k}_{\rho })$ satisfies (21) truncated on a compact domain $S_{\bar{x}} \subset {\mathbb {Z}}_{\ge }$; hence, it is bounded and has bounded time-derivatives for all $(\varvec{\bar{\kappa }}, \textbf{k}_{\rho }) \in {\mathbb {K}}$ and $t \in [0,T]$. It then follows from (19) that there exist vectors $p_1(t; \, \varvec{\bar{\kappa }}, \textbf{k}_{\rho })$ and $p_2(t; \, \varvec{\bar{\kappa }}, \textbf{k}_{\rho })$ which are bounded and have bounded time-derivatives. Hence, $\Vert r_{\varepsilon } (t; \, \varvec{\bar{\kappa }}, \textbf{k}_{\rho }) \Vert _{l_1(\mathbb {S}_{\bar{x}} \times \mathbb {S}_{y})} = \mathcal {O}(\varepsilon ^{1/2})$ as $\varepsilon \rightarrow 0$ for all $(\varvec{\bar{\kappa }}, \textbf{k}_{\rho }) \in {\mathbb {K}}$ and $t \in [0,T]$ which, together with (23), implies (22).

*Remark*. Constant $c = c({\mathbb {K}}, \mathbb {S}_{\bar{x}} \times \mathbb {S}_{y}, T)$ appearing in (22) increases linearly with *T* (see (26)), i.e. condition $\varepsilon \ll 1/T^2$ is sufficient for achieving accuracy for all $t \in [0, T]$. In Sect. 3, we demonstrate with numerical simulations that, while sufficient, the condition $\varepsilon \ll 1/T^2$ is not necessary for the error to satisfy a bound of the form (22). In particular, we present an example biochemical network for which $\lim _{t \rightarrow \infty } \Vert p_{\varepsilon }(\cdot ,\cdot ,t) - p_0(\cdot ,\cdot ,t) \Vert _{l_1( \mathbb {S}_{\bar{x}} \times \mathbb {S}_{y})} = \mathcal {O}(\varepsilon ^{1/2})$, showing that even stationary PMFs can obey an error bound of the form (22).

*Remark*. Result (22) remains valid if for all $t \in [0,T]$ the rate coefficients $\varvec{\bar{\kappa }} = \varvec{\bar{\kappa }}(t)$ and $\textbf{k}_{\rho } = \textbf{k}_{\rho }(t)$ are nonnegative-valued functions of time with continuous first derivatives.

*Remark*. One can derive error bounds analogous to (22) for scalings other than (16), which are consistent with (14); however, note that such scalings lead to systems of perturbation equations whose form is not the same as (19), see also Appendix 2. In particular, one can show that, under the fractional-power scaling $\kappa _1 = \bar{\kappa }_1 \varepsilon ^{-n/d}$ and $\kappa _2 = \bar{\kappa }_2 \varepsilon ^{-(1 - n/d)}$, with $n, d \in {\mathbb {Z}}_{>}$ and $n/d < 1$, the error can be asymptotically bounded by $c \varepsilon ^{1/d}$ for some $c > 0$; scaling (16) is a special case obtained by taking $n = 1$ and $d = 2$.

The joint-PMF error estimate (22) holds for every choice of the rate coefficients $\bar{\kappa }_1$ and $\bar{\kappa }_2$, and for every choice of the stoichiometric coefficients $\tilde{\nu }$ and $\tilde{\gamma }$. Under the particular choices (14) and (15), Proposition 2.1 implies the following marginal-PMF error estimate. In what follows, we let $p_{\varepsilon }^{x}(x,t) \equiv \langle 1, p_{\varepsilon }(x,\cdot ,t)\rangle$ be the *x*-marginal PMF of network (2).

#### Corollary 2.1

Consider the input network $\mathcal {R}_0$ (1). Consider also the output network $\mathcal {R}_{\varepsilon }$ (2) with rate coefficients $\kappa _1$ and $\kappa _2$ satisfying (14) and (16), and with stoichiometric coefficients $\tilde{\nu }$ and $\tilde{\gamma }$ satisfying (15). Let $p_{0}(x,t; \, k_1, \textbf{k}_{\rho })$ be the PMF of $\mathcal {R}_0$, and $p_{\varepsilon }(x,y,t; \, \varvec{\bar{\kappa }}, \textbf{k}_{\rho })$ be the PMF of $\mathcal {R}_{\varepsilon }$. Assume that $p_{\varepsilon }(x,y,0; \, \varvec{\bar{\kappa }}, \textbf{k}_{\rho }) = p_{0}(x,0; \, k_1, \textbf{k}_{\rho }) \delta _{y,0}$. Then, for every ${\mathbb {K}} \subset \mathbb {R}_{>}^{M+1}$, $\mathbb {S}_{x} \times \mathbb {S}_{y} \subset {\mathbb {Z}}_{\ge }^{2}$, where $\mathbb {S}_y \supseteq [0,\text {max} \{2, \tilde{\gamma }\}]$, and [0, *T*], where $T> 0$, there exist constants $c > 0$ and $\varepsilon _0> 0$ such that for all $(\varvec{\bar{\kappa }}, \textbf{k}_{\rho })^{\top } \in {\mathbb {K}}$, $t \in [0,T]$ and $\varepsilon \in (0, \varepsilon _0]$ the *x*-marginal PMF $p_{\varepsilon }^{x}(x,t; \, \varvec{\bar{\kappa }}, \textbf{k}_{\rho })$ satisfies

27 $$\begin{aligned} \left\| p_{\varepsilon }^{x}(\cdot ,t; \, \varvec{\bar{\kappa }}, \textbf{k}_{\rho }) - p_0(\cdot ,t; \, k_1, \textbf{k}_{\rho }) \right\| _{l_1(\mathbb {S}_x)}&\le c \, \varepsilon ^{1/2}. \end{aligned}$$

#### Proof

If the conditions (14), (15) and (16) hold, then the effective CME of the output network (2), given by (21), is identical to the CME of the input network (1), given by (13). The marginal-PMF error bound (27) follows from its joint-PMF counterpart (22).

*Remark*. The assumption $p_{\varepsilon }(x,y,0; \, \varvec{\bar{\kappa }}, \textbf{k}_{\rho }) = p_{0}(x,0; \, k_1, \textbf{k}_{\rho }) \delta _{y,0}$ can be relaxed under a suitable initial-layer analysis, which we do not pursue in this paper.

*Remark*. The *y*-marginal PMF $p_{\varepsilon }^{y}(y,t) \equiv \langle 1, p_{\varepsilon }(\cdot ,y,t)\rangle$ of $\mathcal {R}_{\varepsilon }$ obeys an analogous error bound:

28 $$\begin{aligned} \left\| p_{\varepsilon }^{y}(\cdot ,t; \, \varvec{\bar{\kappa }}, \textbf{k}_{\rho }) - \delta _{y,0} \right\| _{l_1(\mathbb {S}_y)}&\le c \, \varepsilon ^{1/2}. \end{aligned}$$

### Multi-reaction approximations

The results from Sects. (2.1) and (2.2) have been achieved when a single input tri-molecular reaction  is replaced by the output bi-molecular network $\mathcal {R}_{1}^{\varepsilon }(X; \, Y, \kappa _1) \cup \mathcal {R}_{2}(X; \, Y, \kappa _2, \tilde{\nu }, \tilde{\gamma })$ involving one auxiliary species *Y*. Assume now that we wish to approximate multiple input tri-molecular reactions, , where $2 \le M' \le M$. Then, performing analogous perturbation analysis as in Sects. (2.1)–(2.2), one can readily prove that analogous convergence results hold if the $M'$ input reactions are replaced by $3 M'$ output reactions $\bigcup _{j = 1}^{M'} (\mathcal {R}_{1}^{\varepsilon }(X; \, Y_j, \kappa _1^j) \cup \mathcal {R}_{2}(X; \, Y_j, \kappa _2^j, \tilde{\nu }_j, \tilde{\gamma }_j))$ involving $M'$ auxiliary species $\{Y_j\}_{j = 1}^{M'}$. More efficiently, one can readily prove that the same convergence results also hold if the $M'$ input reactions are replaced by $(M' + 2)$ output reactions $\mathcal {R}_{1}^{\varepsilon }(X; \, Y, \kappa _1) \bigcup _{j = 1}^{M'} \mathcal {R}_{2}(X; \, Y, \kappa _2^j, \tilde{\nu }_j, \tilde{\gamma }_j)$ involving only one auxiliary species *Y*.

#### Example 2.1

Consider the third-order input network29By applying the algorithm (2) independently to each of the two reactions from (29), one obtains the second-order output network30The kinetic and stoichiometric conditions (14) and (15) are respectively given by:

31 $$\begin{aligned} \varepsilon \kappa _1^1 \kappa _2^1&= k_1, \varepsilon \kappa _1^2 \kappa _2^2 = k_2, \nonumber \\ \tilde{\nu }_{1}&= 4 - 2 \tilde{\gamma }_1, \tilde{\nu }_{2} = 2 - 2 \tilde{\gamma }_2. \end{aligned}$$

Output network (30) contains 6 reactions and 2 auxiliary species $Y_1$ and $Y_2$. More efficiently, an alternative output network is given by32and contains 4 reactions and 1 auxiliary species *Y*. The kinetic and stoichiometric conditions read

33 $$\begin{aligned} \varepsilon \kappa _1 \kappa _2^1&= k_1, \varepsilon \kappa _1 \kappa _2^2 = k_2, \nonumber \\ \tilde{\nu }_{1}&= 4 - 2 \tilde{\gamma }_1, \tilde{\nu }_{2} = 2 - 2 \tilde{\gamma }_2. \end{aligned}$$

## Example: The Schlögl network

In this section, we apply the results developed in Sect. 2 to the one-species third-order Schlögl network (Schlögl 1972), given by34where $\varnothing$ represents species or processes that are not explicitly modelled. In Fig. a, we display as a black curve the stationary PMF $p_0 = p_0(x; \, k_1, (k_2, k_3, k_4))$ for the input network (34) under a particular choice of the rate coefficients, which has been obtained by numerically solving the underlying stationary CME; one can notice that $p_0$ displays two maxima (bi-modality). Approximating the third-order reaction  according to (2), while preserving the residual network , one obtains:35The stoichiometric condition (15) demands that $\tilde{\nu } = (2 - 2 \tilde{\gamma })$, and there are two choices: taking $\tilde{\gamma } = 0$ implies that $\tilde{\nu } = 2$, taking $\tilde{\gamma } = 1$ implies that $\tilde{\nu } = 0$, while taking $\tilde{\gamma } \ge 2$ implies that $\tilde{\nu } < 0$, which is biochemically infeasible. In what follows, we take $(\tilde{\nu },\tilde{\gamma }) = (0,1)$, and consider different scaling factors *s* to satisfy the kinetic condition (14).

**Fig. 1:**
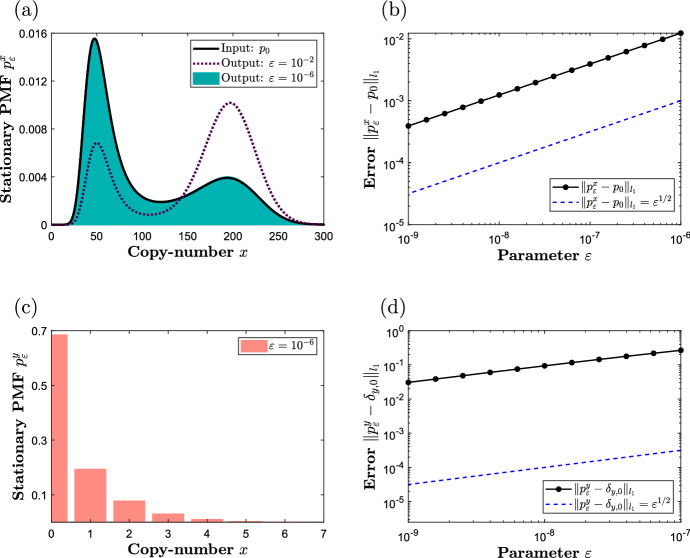
Panel **a** displays the stationary PMF of the input network (34) as a black curve, and the *x*-marginal PMF for the output network  (35), under (36), for different values of the asymptotic parameter $\varepsilon$. Panel **b** displays as black dots, interpolated with the solid lines, a log-log plot of the $l_1$-distance between the PMFs for networks (34) and (35) as a function of $\varepsilon$. Analogous plots are shown in panels **c** and **d** for the *y*-marginal PMF for (35). The parameters are fixed to $(k_1,k_2,k_3,k_4) = (10^{-3}, 1125,37.5,0.36)$ and $(\tilde{\nu },\tilde{\gamma }) = (0,1)$; the plots have been obtained by numerically solving the underlying stationary CMEs

Let us first satisfy (14) by setting

36 $$\begin{aligned} s = 1/2, \;\; \bar{\kappa }_1 = \bar{\kappa }_2 = k_1^{1/2}. \end{aligned}$$

**Fig. 2:**
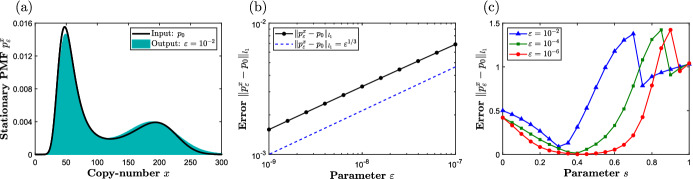
Panel **a** displays the stationary PMF of the input network (34) as a black curve, and the *x*-marginal PMF for the output network  (35), under (37) with $\varepsilon = 10^{-2}$, as a blue histogram. Panel **b** displays a log-log plot of the $l_1$-distance between the PMFs for networks (34) and (35) as a function of $\varepsilon$. Panel **c** displays the error as a function of the scaling factor *s* for three different values of $\varepsilon$, and with $(\bar{\kappa }_1, \bar{\kappa }_2)$ and $(k_1, k_2, k_3, k_4)$ fixed as in Fig. 1

In Fig. 1a, we display the stationary *x*-marginal PMF of the output network (35) under (36), denoted by $p_{\varepsilon }^x = p_{\varepsilon }^x(x; \, (\bar{\kappa }_1, \bar{\kappa }_2), (k_2, k_3, k_4))$, for different values of the parameter $\varepsilon$. In particular, when $\varepsilon = 10^{-2}$, the PMF is shown as a dashed purple curve; while bi-modal, this intermediate PMF is inaccurately distributed. On the other hand, when $\varepsilon = 10^{-6}$, the PMF $p_{\varepsilon }^x$ is shown as a blue histogram, and is in an excellent match with target PMF $p_0$. In Fig. 1b, we show a log-log plot of a numerically approximated error $\Vert p_{\varepsilon }^x - p_0\Vert _{l_1}$ as a function of $\varepsilon$. Also shown, as a dashed blue line, is the reference curve $\Vert p_{\varepsilon }^x - p_0 \Vert _1 = \varepsilon ^{1/2}$; one can notice an excellent match in the slopes of the two curves, in accordance with the finite-time result (27) from Corollary 2.1. In Fig. c, we display the stationary *y*-marginal PMF for network (35) when $\varepsilon = 10^{-6}$, which is shown in Fig. 2d to converge to the Kronecker-delta function centered at zero in accordance with the finite-time result (28).

Corollary 2.1 provides information about the error $\Vert p_{\varepsilon }^x - p_0\Vert _{l_1}$ in the limit $\varepsilon \rightarrow 0$. Let us now discuss how one may decrease this error for a fixed $\varepsilon$ by choosing an appropriate scaling factor *s*. To this end, note that network (2) consists of an ordered chain of reactions: in order for $\mathcal {R}_2$ to fire, and mimic (1), one requires that the forward reaction from $\mathcal {R}_1^{\varepsilon }$ fires first. The reactant of the forward reaction from $\mathcal {R}_1^{\varepsilon }$, forming the start of the chain, is given by 2*X*, and the propensity function is given by $\alpha _1(x) = \kappa _1 x (x - 1)$. On the other hand, $\mathcal {R}_2$ involves as a reactant the auxiliary species *Y*, with the propensity function $\alpha _2(x,y_1) = \kappa _2 x y$. Since *Y*(*t*) spends most of the time at $y = 0$ for sufficiently small $\varepsilon$, it follows that the underlying joint-PMF is concentrated in the neighborhood of the *x*-axis, and $\alpha _2(x,y)/\kappa _2 < \alpha _1(x)/\kappa _1$. This observation suggests that, for a fixed smaller $\varepsilon$, there is an optimal ratio $\kappa _1/\kappa _2$, sufficiently small to speed up reaction $\mathcal {R}_2$, and sufficiently large to ensure that $\mathcal {R}_1^{\varepsilon }$ is triggered often enough. To this end, let us now satisfy (14) by setting

37 $$\begin{aligned} s = 1/3, \;\; \bar{\kappa }_1 = \bar{\kappa }_2 = k_4^{1/2}. \end{aligned}$$

In Fig. 2a, we display the stationary *x*-marginal PMF of the output network (35) under (37) when $\varepsilon = 10^{-2}$. Comparing Figs. 1a and 2a, one can notice that the scaling (37) leads to a significantly better approximation than (36) when $\varepsilon = 10^{-2}$. However, comparing Figs. 1b and 2b, one can notice that the convergence under the scaling (37) is slower than under (36), with the former occurring at a rate $\varepsilon ^{1/3}$; see also a remark below Proposition 2.1. In Fig. 2c, we display the $l_1$-distance between the input and output PMFs as a function of the scaling factor *s* for three different values of the parameter $\varepsilon$. One can notice that the error appears to be minimized approximately at $s = 3/10$ when $\varepsilon = 10^{-2}$, and that, for larger values of $\varepsilon$, an overall better performance is achieved by taking $s < 1/2$. Figure 2c also suggests that the error does not converge to zero at the degenerate points $s = 0$ and $s = 1$.

## General case: Multi-species higher-order reactions

Let us consider an arbitrary input mass-action reaction network $\mathcal {R}_0 = \mathcal {R}_0(\mathcal {X})$ involving *N* biochemical species $\mathcal {X} = \{X_1, X_2, \ldots , X_N\}$, given by

38 $$\begin{aligned} \mathcal {R}_0&= \left( \sum _{i = 1}^{m} \nu _{1,i} X_{i} \xrightarrow []{k_1} \sum _{i = 1}^N \bar{\nu }_{1,i} X_ i \right) \cup \mathcal {R}_{\rho }, \; \; \text {where } \{\nu _{1,i} \in {\mathbb {Z}}_{>}\}_{i = 1}^m \text { and } \sum _{i = 1}^{m} \nu _{1,i} = n \ge 3, \nonumber \\ \mathcal {R}_{\rho }(\mathcal {X})&= \bigcup _{j = 2}^M \left( \sum _{i = 1}^{N} \nu _{j,i} X_{i} \xrightarrow []{k_j} \sum _{i = 1}^N \bar{\nu }_{j,i} X_ i \right) . \end{aligned}$$

In particular, we assume that the first reaction involves $m \ge 1$ distinct reactants $\{X_1, X_2, \ldots , X_m\} \subseteq \mathcal {X}$, which we enforce by demanding that the underlying reactant stoichiometric coefficients are nonzero; we also assume that the first reaction is of order $n \ge 3$. Furthermore, for convenience, we assume that the reactant species in the first reaction are ordered according to nondecreasing stoichiometric coefficients, i.e. $\nu _{1,i} \le \nu _{1,j}$ if $i < j$, for all $i, j \in \{1, 2, \ldots , m\}$. We wish to approximate the *n*-th order target (first) reaction from (38) with a suitable second-order network, while leaving the residual network $\mathcal {R}_{\rho }$ unchanged, analogously as in Sect. 2. To this end, consider the output mass-action reaction network $\mathcal {R}_{\varepsilon } = \mathcal {R}_{\varepsilon }(\mathcal {X}, \mathcal {Y})$ given by

39 $$\begin{aligned} \mathcal {R}_{\varepsilon }&= {\left\{ \begin{array}{ll} \Big (\mathcal {R}_{1}^{\varepsilon }(X_1,X_1) \, \bigcup _{i = 2}^{n - 2} \mathcal {R}_{i}^{\varepsilon }(X_1) \, \cup \, \mathcal {R}_{n-1}(X_1) \Big ) \cup \mathcal {R}_{\rho }(\mathcal {X}), &{} \text {if } m = 1, \\ \Big (\mathcal {R}_{1}^{\varepsilon }(X_1, X_2) \, \bigcup _{i = 2}^{\nu _{1,1}} \mathcal {R}_{i}^{\varepsilon }(X_1) \, \bigcup _{l = 2}^{m-1} \bigcup _{i = \delta _{l,2} + \sum _{j=1}^{l-1} \nu _{1,j}}^{-1 + \sum _{j=1}^{l} \nu _{1,j}} \mathcal {R}_{i}^{\varepsilon }(X_l) \\ \, \bigcup _{i = \delta _{m,2} + \sum _{j=1}^{m-1} \nu _{1,j}}^{n - 2} \mathcal {R}_{i}^{\varepsilon }(X_{m}) \, \cup \,\mathcal {R}_{n-1}(X_m) \Big ) \cup \mathcal {R}_{\rho }(\mathcal {X}), &{} \text {if } m \ge 2, \end{array}\right. } \end{aligned}$$

with the convention that $\bigcup _{l = a}^{b} \mathcal {R}(l) = \emptyset$ if $a > b$, where $\emptyset$ is the empty set, and with the sub-networks40Network $\mathcal {R}_{\varepsilon }(\mathcal {X}, \mathcal {Y})$, given by (39)–(40), contains $(n-2)$ auxiliary species $\mathcal {Y} = \{Y_1, Y_2, \ldots , Y_{n-2} \}$, and consists of $(2 n - 3)$ reactions, $(n-2)$ of which are first-order, and $(n-1)$ of second-order. Reaction network (2) from Sect. 2 is a special case of (39)–(40) with $m = N = 1$ and $n = 3$.

### Kinetic and stoichiometric conditions

In Appendix 2, we generalize the formal perturbation analysis from Sect. 2.1 and derive kinetic and stoichiometric conditions, analogous to (14) and (15), respectively, which ensure that the CMEs for the input network (38) and the output network (39)–(40) match. In particular, the generalized kinetic condition is given by

41 $$\begin{aligned} \varepsilon ^{n-2} \prod _{i = 1}^{n-1} \kappa _i&= k_1, \; \; \; \text {where } \kappa _1, \kappa _2, \ldots , \kappa _{n-1} = o(\varepsilon ^{-1}) \; \; \text {as } \varepsilon \rightarrow 0. \end{aligned}$$

Requirement (41) states that the product of the rate coefficients of the slower reactions from (40), $\prod _{i = 1}^{n-1} \kappa _i$, divided by the product of the rate coefficients of the faster reactions, $1/\varepsilon ^{n-2}$, must be equal to the rate coefficient of the target reaction from (38), $k_1$. On the other hand, when the reaction $\mathcal {R}_{n-1}(X_j)$ from (40) does not contain the auxiliary species $\{Y_1, Y_2, \ldots , Y_{n-3}\}$, then the generalized stoichiometric conditions are given by

42 $$\begin{aligned} \tilde{\nu }_i&= \bar{\nu }_{1,i} - (\nu _{1,i} - \delta _{i,m}) \tilde{\gamma }_{n-2}, \; \; \text {for all } i \in \{1, 2, \ldots , m \}, \; \; \; \text {and } (\tilde{\gamma }_1, \tilde{\gamma }_2, \ldots , \tilde{\gamma }_{n-3}) = (0, 0, \ldots , 0). \end{aligned}$$

The stoichiometric conditions valid when $(\tilde{\gamma }_1, \tilde{\gamma }_2, \ldots , \tilde{\gamma }_{n-3}) \ne \textbf{0}$ take a more complicated form, and can be obtained algebraically as explained in Appendix 2. One can also readily obtain the stoichiometric conditions graphically, as we now outline via an example.

#### Example 4.1

Consider the input reaction

43 $$\begin{aligned} \mathcal {R}_0(X_1, X_2,X_3)&= \left( 2 X_1 + 2 X_2 \xrightarrow []{k_1} 4 X_1 + 3 X_2 + X_3 \right) , \end{aligned}$$

which contains $N = 3$ species $\mathcal {X} = \{X_1, X_2, X_3\}$, $m = 2$ distinct reactants $\{X_1, X_2\}$, and is of order $n = 4$. The reactant and product stoichiometric vectors are given by $(\nu _{1,1}, \nu _{1,2}, \nu _{1,3})^{\top } = (2, 2,0)^{\top }$ and $(\bar{\nu }_{1,1}, \bar{\nu }_{1,2}, \bar{\nu }_{1,3})^{\top } = (4, 3,1)^{\top }$, respectively, and the reaction vector reads $(\Delta x_{1,1}, \Delta x_{1,2},\Delta x_{1,3})^{\top } = (4,3,1)^{\top } - (2,2,0)^{\top } = (2,1,1)^{\top }$. Output network (39)–(40) takes the form $\mathcal {R}_{\varepsilon } = \mathcal {R}_{1}^{\varepsilon }(X_1,X_2) \cup \mathcal {R}_{2}^{\varepsilon }(X_1) \cup \mathcal {R}_{3}(X_2)$, with44*Algebraic approach*. Stoichiometric conditions required for matching reaction (43) and $\mathcal {R}_{3}(X_2)$ from (44) can be obtained algebraically from the conservation laws that are locally valid for the fastest two reactions from (44):

45 $$\begin{aligned} \bar{x}_1&= x_1 + y_1 + 2 y_2, \; \; \bar{x}_2 = x_2 + y_1 + y_2. \end{aligned}$$

Applying the difference operator $\Delta$ on (45), using $(\Delta x_1, \Delta x_2, \Delta y_1, \Delta y_2)^{\top } = (\tilde{\nu }_1, \tilde{\nu }_2-1, \tilde{\gamma }_1, \tilde{\gamma }_2 - 1)^{\top }$, and imposing the matching condition $(\Delta \bar{x}_1, \Delta \bar{x}_2)^{\top } = (\Delta x_{1,1}, \Delta x_{1,2})^{\top } = (2,1)^{\top }$, one obtains the stoichiometric conditions

46 $$\begin{aligned} \tilde{\nu }_1&= 4 - (\tilde{\gamma }_1 + 2 \tilde{\gamma }_2), \; \; \tilde{\nu }_2 = 3 - (\tilde{\gamma }_1 + \tilde{\gamma }_2). \end{aligned}$$

*Graphical approach*. Stoichiometric conditions (46) can also be obtained graphically. In particular, fixing $(\tilde{\gamma }_{1}, \tilde{\gamma }_{2})^{\top } = (0, 0)^{\top }$, it follows from (42) that $(\tilde{\nu }_1, \tilde{\nu }_2)^{\top } = (\bar{\nu }_{1,1}, \bar{\nu }_{1,2})^{\top }$, i.e. reaction (43) and $\mathcal {R}_{3}(X_2)$ from (44) have identical products:

47 $$\begin{aligned} \mathcal {R}_{3}(X_2)&= \left( X_2 + Y_2 \xrightarrow []{\kappa _3} 4 X_1 + 3 X_2 + X_3\right) , \text {if } (\tilde{\gamma }_{1}, \tilde{\gamma }_{2}) = (0, 0). \end{aligned}$$

One can now add the formal equalities $\varnothing \doteq (Y_1 - X_1 - X_2)$ (obtained from $Y_1 \doteq (X_1 + X_2)$) and $\varnothing \doteq (Y_2 - 2 X_1 - X_2)$ (obtained from $Y_2 \doteq (X_1 + Y_1) \doteq (2 X_1 + X_2)$) to the products in (47) as many times as desired, as long as the resulting complex contains nonnegative stoichiometric coefficients. For example, by adding the complex $\varnothing \doteq (Y_2 - 2 X_1 - X_2)$ to (47), one obtains

48 $$\begin{aligned} \mathcal {R}_{3}(X_2)&= \left( X_2 + Y_2 \xrightarrow []{\kappa _3} 2 X_1 + 2 X_2 + X_3 + Y_2 \right) , \text {if } (\tilde{\gamma }_{1}, \tilde{\gamma }_{2}) = (0, 1). \end{aligned}$$

Adding the complex $\varnothing \doteq (Y_1 - X_1 - X_2)$ three times to (47) leads to

49 $$\begin{aligned} \mathcal {R}_{3}(X_2)&= \left( X_2 + Y_2 \xrightarrow []{\kappa _3} X_1 + X_3 + 3 Y_1 \right) , \text {if } (\tilde{\gamma }_{1}, \tilde{\gamma }_{2}) = (3, 0), \end{aligned}$$

while adding $\varnothing \doteq (Y_1 - X_1 - X_2)$ twice, and $\varnothing \doteq (Y_2 - 2 X_1 - X_2)$ once, results in

50 $$\begin{aligned} \mathcal {R}_{3}(X_2)&= \left( X_2 + Y_2 \xrightarrow []{\kappa _3} X_3 + 2 Y_1 + Y_2 \right) , \text {if } (\tilde{\gamma }_{1}, \tilde{\gamma }_{2}) = (2, 1). \end{aligned}$$

On the other hand, adding $\varnothing \doteq (Y_2 - 2 X_1 - X_2)$ three times to the products in (47) leads to

51 $$\begin{aligned} \mathcal {R}_{3}(X_2)&= \left( X_2 + Y_2 \xrightarrow []{\kappa _3} - 2 X_1 + X_3 + 3 Y_2 \right) , \text {if } (\tilde{\gamma }_{1}, \tilde{\gamma }_{2}) = (0, 3), \end{aligned}$$

which is not a biochemical reaction, as the product complex is not nonnegative.

*Remark*. The graphical approach taken in Example 4.1 applies generally: one can extract the formal equalities, such as $\varnothing \doteq (Y_1 - X_1 - X_2)$ and $\varnothing \doteq (Y_2 - 2 X_1 - X_2)$, directly from the fastest reactions in $\mathcal {R}_{\varepsilon }$. Writing the final reaction from $\mathcal {R}_{\varepsilon }$ with the same product complex as in the first reaction from $\mathcal {R}_{0}$, one can then add the formal equalities as many times as desired to the products of the final reaction from $\mathcal {R}_{\varepsilon }$, provided the resulting complex remains nonnegative.

### Convergence

Let us now generalize Corollary 2.1 by establishing convergence when the slower rate coefficients from (39)–(40) are all scaled identically:

52 $$\begin{aligned} \kappa _i&= \varepsilon ^{-(n-2)/(n-1)} \bar{\kappa }_i, \; \; \; \; \text {for all } i \in \{1, 2, \ldots , n-1\}, \end{aligned}$$

where $\{\bar{\kappa }_i \}_{i = 1}^{n-1}$ are $\varepsilon$-independent parameters. In what follows, we let $\textbf{x} = (x_1, x_2, \ldots , x_N)^{\top } \in {\mathbb {Z}}_{\ge }^N$, $\textbf{y} = (y_1, y_2, \ldots , y_{n-2})^{\top } \in {\mathbb {Z}}_{\ge }^{n-2}$, $\varvec{\bar{\kappa }} = (\bar{\kappa }_1, \bar{\kappa }_2, \ldots , \bar{\kappa }_{n-1})^{\top } \in \mathbb {R}_{>}^{n-1}$, $\textbf{k}_{\rho } = (k_2, k_3, \ldots , k_M)^{\top } \in \mathbb {R}_{>}^{M-1}$, and $\delta _{\textbf{y},\textbf{0}} = \prod _{i=1}^{n-2} \delta _{y_i,0}$.

#### Theorem 4.1

Consider the input network $\mathcal {R}_0$ (38). Consider also the output network $\mathcal {R}_{\varepsilon }$ (39)–(40), with rate coefficients $\{\kappa _i\}_{i = 1}^{n-1}$ satisfying (41) and (52), and with stoichiometric coefficients $\{\tilde{\nu }_i\}_{i = 1}^m$ and $\{\tilde{\gamma }_i\}_{i = 1}^{n-2}$ satisfying (42). Let $p_{0}(\textbf{x},t; \, k_1, \textbf{k}_{\rho })$ be the PMF of $\mathcal {R}_0$, and $p_{\varepsilon }(\textbf{x}, \textbf{y}, t; \, \varvec{\bar{\kappa }}, \textbf{k}_{\rho })$ be the PMF of $\mathcal {R}_{\varepsilon }$. Assume that $p_{\varepsilon }(\textbf{x}, \textbf{y}, 0; \, \varvec{\bar{\kappa }}, \textbf{k}_{\rho }) = p_{0}(\textbf{x},0; \, k_1, \textbf{k}_{\rho }) \delta _{\textbf{y},\textbf{0}}$. Then, there exists a constant $C \in {\mathbb {Z}}_{>}$ such that for every compact parameter set ${\mathbb {K}} \subset \mathbb {R}_{>}^{M + n - 2}$, compact state-space $\mathbb {S}_{x} \times \mathbb {S}_{y} \subset {\mathbb {Z}}_{\ge }^{N + (n - 2)}$, where $\mathbb {S}_{y} \supseteq [0, C]^{n-2}$, and compact time-interval [0, *T*], where $T> 0$, there exist constants $c > 0$ and $\varepsilon _0 > 0$ such that for all $(\varvec{\bar{\kappa }}, \textbf{k}_{\rho })^{\top } \in {\mathbb {K}}$, $t \in [0,T]$ and $\varepsilon \in (0, \varepsilon _0]$ the $\textbf{x}$-marginal PMF of $\mathcal {R}_{\varepsilon }$, denoted by $p_{\varepsilon }^{x}(\textbf{x},t; \, \varvec{\bar{\kappa }}, \textbf{k}_{\rho })$, satisfies

53 $$\begin{aligned} \left\| p_{\varepsilon }^{x}(\cdot ,t; \, \varvec{\bar{\kappa }}, \textbf{k}_{\rho }) - p_0(\cdot ,t; \, k_1, \textbf{k}_{\rho }) \right\| _{l_1(\mathbb {S}_{x})}&\le c \, \varepsilon ^{1/(n-1)}. \end{aligned}$$

#### Proof

See Appendix 3.

*Remark*. Theorem 4.1 also holds under more general choices of $\{\tilde{\nu }_i\}_{i = 1}^m$ and $\{\tilde{\gamma }_i\}_{i = 1}^{n-2}$, which have been outlined in Example 4.1; see also Appendices 2 and 3. In particular, the fact that a given higher-order input network may be approximated by a variety of different second-order output networks is favorable, since there is a greater flexibility to meet various biochemical constraints which may be necessary for successful experimental implementations; see also Example 4.2.

*Remark*. Algorithm (39)–(40) and Theorem 4.1 extend naturally to the case when multiple higher-order input reactions are approximated by second-order ones; see Sections 2.3 and 5.2.

*Remark*. Analogous remarks as those under Proposition 2.1 also apply to Theorem 4.1. In particular, constant $c = c({\mathbb {K}}, \mathbb {S}_{x} \times \mathbb {S}_{y} , T)$ from (53) increases linearly with time *T*, so that a sufficient condition for achieving accuracy for all $t \in [0, T]$ is that $\varepsilon \ll 1/T^{n-1}$; hence, to meet this sufficient condition for a fixed *T*, the higher the order of the target reaction, *n*, the smaller the asymptotic parameter, $\varepsilon$, must be chosen. Furthermore, note that (53) remains valid if for all $t \in [0,T]$ the rate coefficients $\varvec{\bar{\kappa }} = \varvec{\bar{\kappa }}(t)$ and $\textbf{k}_{\rho } = \textbf{k}_{\rho }(t)$ are continuously differentiable nonnegative-valued functions of time.

To formulate Theorem 4.1, we have assumed a fixed ordering of the reactants and reactions in (39)–(40). One can readily prove analogous results for other suitable orderings.

#### Example 4.2

Consider the third-order input reaction

54 $$\begin{aligned} \mathcal {R}_0(X_1, X_2)&= \left( X_1 + 2 X_2 \xrightarrow []{k_1} \varnothing \right) . \end{aligned}$$

Output network (39)–(40) is given by $\mathcal {R}_{\varepsilon } = \mathcal {R}_{1}^{\varepsilon }(X_1,X_2) \cup \mathcal {R}_{2}(X_2)$, where55in particular, the forward reaction from $\mathcal {R}_{1}^{\varepsilon }$ is a second-order hetero-reaction, involving two distinct reactants $X_1$ and $X_2$. One can readily show that the results presented in this section also hold for the output network $\mathcal {R}_{1}^{\varepsilon }(X_2,X_2) \cup \mathcal {R}_{2}(X_1)$, given by56for which the forward reaction from $\mathcal {R}_{1}^{\varepsilon }$ is a second-order homo-reaction, involving only $X_2$ as reactants. Another admissible output network is $\mathcal {R}_{1}^{\varepsilon }(X_1, \varnothing ) \cup \mathcal {R}_{2}^{\varepsilon }(X_2) \cup \mathcal {R}_{3}(X_2)$, given by57for which the forward reaction from $\mathcal {R}_{1}^{\varepsilon }$ is of first-order.

Using analogous perturbation analysis to that underpinning Theorem 4.1, one can show that the $(x_1, x_2)$-marginal PMF of (56) also converges to the PMF of (54) at a rate $\varepsilon ^{1/2}$ as $\varepsilon \rightarrow 0$; the same convergence occurs for the output network (57) at a rate $\varepsilon ^{1/3}$, since two, and not only one, auxiliary species are introduced. Depending on the application area, a particular output network might be more desirable than others; for example, given a set of molecular species $\{X_1, X_2, Y_1\}$ with predefined biophysical properties, it may be easier to experimentally realize a reaction of the form $2 X_2 \rightarrow Y_1$ than $X_1 + X_2 \rightarrow Y_1$.

## Examples: Noise-induced phenomena

In this section, we apply the results from Sect. 4 to two test networks arising from theoretical synthetic biology and displaying noise-induced phenomena that are absent at the deterministic level. In particular, the first network, given by (58), plays an important role in the stochastic morpher controller (Plesa et al. 2021) that can globally morph the PMF of a suitable reaction network into any desired form. The second network, given by (63), is part of the noise-control algorithm (Plesa et al. 2018) that can redesign a given reaction network to locally reshape the underlying PMF in a mean-preserving manner.

### Biochemical Kronecker-delta function

Let us consider the fourth-order mass-action input reaction network

58 $$\begin{aligned} \mathcal {R}_0(X)&= \left( \varnothing \xrightarrow []{k_1} X \right) \cup \left( 4 X \xrightarrow []{k_2} 3 X \right) . \end{aligned}$$

Long-time PMF of (58), under a particular choice of the rate coefficients $k_1 < k_2$, is shown in Fig. a as black dots interpolated with solid lines. The PMF is close to the Kronecker-delta function centered at $x = 3$. In particular, when there are less than four molecules of *X* present, $x < 4$, only the first reaction from (58) fires and *X* experiences a constant positive drift until four molecules are present. When $x \ge 4$, both reactions from (58) fire, with the second one, having a larger propensity function, overpowering the first one and generating a net-negative drift. The combined effect of the two reactions forces *X* to spend most of the time at the state $x = 3$.

Applying the algorithm (39)–(40) on the fourth-order input network (58), one obtains a suitable second-order output network given by59The stoichiometric condition (42) for network (59) with $\tilde{\gamma }_1 = 0$ is given by

60 $$\begin{aligned} \tilde{\nu }&= 3 - 3 \tilde{\gamma }_2; \end{aligned}$$

we fix $\tilde{\gamma }_2 = 1$, so that $\tilde{\nu } = 0$. On the other hand, the kinetic condition (41) for (59) reads

61 $$\begin{aligned} \varepsilon ^2 \kappa _1 \kappa _2 \kappa _3&= k_{2}, \; \; \; \text {where } \kappa _1, \kappa _2, \kappa _3 = o(\varepsilon ^{-1}) \; \; \text {as } \varepsilon \rightarrow 0, \end{aligned}$$

which, using (52) with e.g. $(\bar{\kappa }_1, \bar{\kappa }_2, \bar{\kappa }_3) = (k_2, 1, 1)$, is satisfied with

62 $$\begin{aligned} \kappa _1&=k_2 \varepsilon ^{-2/3}, \; \; \kappa _2 = \varepsilon ^{-2/3}, \; \; \kappa _3 = \varepsilon ^{-2/3}. \end{aligned}$$

**Fig. 3:**
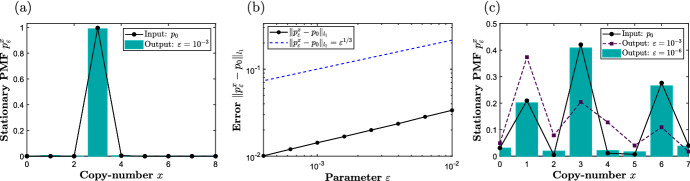
Panel **a** displays the long-time PMF of the input network (58) with $(k_1, k_2) = (10^{-3}, 10^{-2})$ as black dots interpolated with solid lines; the long-time *x*-marginal PMF of the output network (59), with $(\tilde{\nu }, \bar{\gamma }_1,\bar{\gamma }_2) = (0,0,1)$, under rate coefficients (62), is shown as a blue histogram when $\varepsilon = 10^{-3}$. Panel **b** displays a log-log plot of the $l^1$-distance between the long-time PMFs for networks (58) and (59). Panel **c** displays the long-time PMF of the input network (63)–(64) with $(k_1, k_2, k_{2,5}, \tilde{k}_{4,2}) = (1,1,1,1)$, and the *x*-marginal PMF of the output network (65)–(66), under rate coefficients (68) with $\beta = 1/12$, for two different values of $\varepsilon$. The conservation constant for (63) is fixed to $c = 7$, and (65) is initialized with zero copy-numbers of the species $\{Y_i\}_{i = 1}^5$ and $\tilde{Y}_4$. The plots have been generated using the Gillespie algorithm (Gillespie 1977)

In Fig. 3a, we display the long-time *x*-marginal PMF of the output network (59) with $(\tilde{\nu }, \bar{\gamma }_1,\bar{\gamma }_2) = (0,0,1)$ and the rate coefficients (62) with $\varepsilon = 10^{-3}$, which is in an excellent agreement with the input PMF. In Fig. 3b, we show that the output PMF converges to the input one at a rate $\varepsilon ^{1/3}$, consistent with Theorem 4.1.

### Noise-induced tri-modality

In this section, we demonstrate how the approach from Sect. 4, developed to approximate a single higher-order target reaction from an input network, generalizes to the case of multiple target input reactions; analogous ideas have been discussed in Sect. 2.3 for third-order reactions. In particular, one can apply the algorithm (39)–(40) to each of the target input reactions independently; however, such an approach may lead to output networks with larger number of reactions and auxiliary species $\mathcal {Y}$, which may be biochemically expensive to engineer. More efficiently, if some of the higher-order input reactions involve common reactant sub-complexes, then some of the intermediate species $\mathcal {Y}$ can be re-used to simultaneously approximate multiple input reactions. To illustrate these ideas, consider the seventh-order input network $\mathcal {R}_0 = \mathcal {R}_0(X_1, X_2)$ given by

63 $$\begin{aligned} \mathcal {R}_0&= \mathcal {R}_{\rho }(X_1, X_2) \cup \mathcal {R}_{2,5}(X_1, X_2) \cup \mathcal {\tilde{R}}_{4,2}(X_1, X_2), \end{aligned}$$

where64Species $X_1$ and $X_2$ from (63) are conserved, $x_1 + x_2 = c$; in what follows, we fix the conservation constant to $c = 7$. Network (63) has been obtained by applying the noise-control algorithm (Plesa et al. 2018) on the residual network $\mathcal {R}_{\rho }$; in particular, sub-networks $\mathcal {R}_{2,5}$ and $\mathcal {\tilde{R}}_{4,2}$, called zero-drift networks, introduce a state-dependent noise and decrease the PMF of $\mathcal {R}_{\rho }$ at $x_1 = 2$ and $x_1 \in \{4,5\}$, respectively, while preserving the underlying mean. In Fig. 3c, we display the long-time PMF of (63) as black dots interpolated with solid lines. One can notice that the network displays noise-induced tri-modality, with the modes $x_1 \in \{1, 3, 6\}$.

Applying algorithm (39)–(40) to each of the four reactions from $\mathcal {R}_{2,5} \cup \mathcal {\tilde{R}}_{4,2}$ independently requires 18 auxiliary species and 40 reactions in total, i.e. 5 auxiliary species and 11 reactions for each of the reaction from $\mathcal {R}_{2,5}$, and 4 auxiliary species and 9 reactions for each of the reaction from $\mathcal {\tilde{R}}_{4,2}$. However, since both reactions from $\mathcal {R}_{2,5}$ involve the same reactants, one can reduce their order simultaneously by using 5 auxiliary species; similarly, 4 auxiliary species suffice to reduce the order of both reactions from $\mathcal {\tilde{R}}_{4,2}$. Furthermore, all of the reactions from $\mathcal {R}_{2,5} \cup \mathcal {\tilde{R}}_{4,2}$ involve a common reactant sub-complex, namely $(2 X_1 + 2 X_2)$, so that, instead of using 9 auxiliary species for $\mathcal {R}_{2,5} \cup \mathcal {\tilde{R}}_{4,2}$, one can use only 6. These considerations give rise to the output network

65 $$\begin{aligned} \mathcal {R}_{\varepsilon }&= \mathcal {R}_{\rho } \cup \left\{ \mathcal {R}_{1}^{\varepsilon }(X_1, X_1), \; \mathcal {R}_{2}^{\varepsilon }(X_2), \; \mathcal {R}_{3}^{\varepsilon }(X_2) \right\} \cup \{\mathcal {R}_{4}^{\varepsilon }(X_2), \; \mathcal {R}_{5}^{\varepsilon }(X_2), \; \mathcal {R}_6(X_1)\} \cup \{\tilde{\mathcal {R}}_{4}^{\varepsilon }(X_1), \; \tilde{\mathcal {R}}_5(X_1)\}, \end{aligned}$$

where66The sub-network $\mathcal {R}_{2,5}$ from (64) is approximated by $\bigcup _{i=1}^5 \mathcal {R}_{i}^{\varepsilon } \cup \mathcal {R}_6$, while $\mathcal {\tilde{R}}_{4,2}$ by $\bigcup _{i=1}^3 \mathcal {R}_{i}^{\varepsilon } \cup \tilde{\mathcal {R}}_{4}^{\varepsilon } \cup \tilde{\mathcal {R}}_5$; sub-network $\left\{ \mathcal {R}_{1}^{\varepsilon }, \mathcal {R}_{2}^{\varepsilon }, \mathcal {R}_{3}^{\varepsilon } \right\}$ encodes the common sub-complex $(2 X_1 + 2 X_2)$. Instead of applying (39)–(40) independently to each of the reaction from $\mathcal {R}_{2,5} \cup \mathcal {\tilde{R}}_{4,2}$, which requires 18 auxiliary species and 40 reactions, we have achieved the same goal in (65) with 6 auxiliary species and 16 reactions.

Network (65)–(66) satisfies the stoichiometric conditions (42), since $\mathcal {R}_6$ and $\tilde{\mathcal {R}}_5$ do not contain any auxiliary species $\mathcal {Y} = \{Y_1, Y_2, Y_3, Y_4, \tilde{Y}_4, Y_5\}$ as products. One can reduce the product stoichiometric coefficients of $X_1$ and $X_2$ in $\mathcal {R}_6$ and $\tilde{\mathcal {R}}_5$ by introducing suitable species $\mathcal {Y}$ as products (see also Example 4.1); for simplicity, we consider the form (66) in this paper. On the other hand, kinetic conditions (41) take the form

67 $$\begin{aligned} \varepsilon ^5 \left( \kappa _1 \kappa _2 \kappa _3 \right) \kappa _4 \kappa _5 \kappa _6&= k_{2,5}, \; \; \; \text {where } \kappa _1, \kappa _2, \ldots , \kappa _6 = o(\varepsilon ^{-1}) \; \; \text {as } \varepsilon \rightarrow 0, \nonumber \\ \varepsilon ^4 \left( \kappa _1 \kappa _2 \kappa _3 \right) \tilde{\kappa }_4 \tilde{\kappa }_5&= \tilde{k}_{4,2}, \; \; \; \text {where } \tilde{\kappa }_4, \tilde{\kappa }_5 = o(\varepsilon ^{-1}) \; \; \text {as } \varepsilon \rightarrow 0. \end{aligned}$$

To achieve a higher accuracy for larger values of $\varepsilon$, we satisfy the kinetic conditions with

68 $$\begin{aligned} \kappa _1&= \left( \varepsilon ^{-\frac{5}{6}} (k_{2,5})^{\frac{1}{6}} \right) \varepsilon ^{5 \beta }, \nonumber \\ \kappa _i&= \left( \varepsilon ^{-\frac{5}{6}} (k_{2,5})^{\frac{1}{6}} \right) \varepsilon ^{-\beta }, \; \; 0 \le \beta < 1/6, \; \; \text {for all } i \in \{2, 3, 4, 5, 6\}, \nonumber \\ \tilde{\kappa }_i&= \varepsilon ^{-2} (\kappa _1 \kappa _2 \kappa _3)^{-\frac{1}{2}} (\tilde{k}_{4,2})^{\frac{1}{2}}, \; \; \text {for all } i \in \{4, 5\}. \end{aligned}$$

In particular, guided by the discussion in Sect. 3 (see also Fig. 2c), we introduce an auxiliary parameter $\beta$ to slow down the reaction with rate coefficient $\kappa _1$, and speed up the remaning ones. In Fig. 3c, we display the long-time *x*-marginal PMF of (65) with rate coefficients (68), with the auxiliary parameter $\beta = 1/12$, for $\varepsilon = 10^{-3}$ and $\varepsilon = 10^{-6}$, the latter of which is in a good agreement with the long-time PMF of the input network (63).

## Discussion

In this paper, we have shown that, by introducing auxiliary species (dimension expansion) and suitable time-scaled-separated reactions, any higher-order input reaction network can be mapped to a second-order output one, with the underlying stochastic dynamics being preserved. This order-reduction algorithm has been previously formally established at the deterministic level for third- and fourth-order reactions (Tyson 1973; Cook et al. 1989; Wilhelm 2000). In this paper, we have generalized this algorithm to reactions with arbitrary number and composition of reactants, and we have augmented our formal results with rigorous convergence analyses at the stochastic level. In particular, we have shown that the time-dependent probability distributions of the input and output networks are arbitrarily close over suitable bounded domains in an appropriate asymptotic limit of some of the underlying rate coefficients.

In Sect. 2, we have shown that an arbitrary one-species input reaction of order $n = 3$, given in (1), can be approximated by a family of second-order output networks, given in (2), provided the kinetic and stoichiometric conditions (14) and (15), respectively, are satisfied. Convergence for a family of the output networks has been proved in Corollary 2.1. In Sect. 3, we have numerically verified the results from Sect. 2 on the Schlögl network (34). In Appendices 2–3, the results from Sect. 2 have been generalized to arbitrary multi-species reactions of order $n \ge 3$, and these results have been presented in Sect. 4. In particular, we have shown that an arbitrary multi-species input reaction of order $n \ge 3$, given in (38), can be approximated with a family of second-order output networks, given in (39)–(40), provided the kinetic and stoichiometric conditions (41) and (42), respectively, hold. Convergence for a particular family of output networks has been established in Theorem 4.1, where we have shown that, for an input reaction of order $n \ge 3$, the order of convergence is given by $(n-1)^{-1}$; hence, the higher the order of the input reaction, the slower the convergence. In Sect. 5, we have applied the results from Sect. 4 to the fourth- and seventh-order input networks (58) and (63), respectively, arising from theoretical synthetic biology (Plesa et al. 2018, 2021), and displaying noise-induced phenomena.

The results established in this paper may play an important role in synthetic biology, and particularly in nucleic-acid-based synthetic biology, also known as DNA computing (Zhang and Winfree 2009). In this context, it has been proved that, assuming one can experimentally vary reaction rate coefficients over a sufficiently large range, any abstract reaction network of up to second-order, under mass-action kinetics, can be experimentally compiled into a physical second-order network with DNA molecules, with the underlying deterministic dynamics being preserved over compact time-intervals (Soloveichik et al. 2010). This molecular compiler has been proved to also preserve the underlying stochastic dynamics (Plesa et al. 2018). In this context, results from Sect. 4, and Theorem 4.1 in particular, imply the following corollary.

### Corollary 6.1

(Universal molecular compiler) Assume that the reaction rate coefficients in the DNA compiler from Soloveichik et al. (2010) can be varied over arbitrarily large range. Then, any mass-action input reaction network, of arbitrary order, can be compiled into a second-order DNA-based output network with the compiler from Soloveichik et al. (2010), in such a way that the probability distributions for the input and output networks are arbitrarily close over sufficiently large compact state-spaces and any compact time-interval.

## Appendix: Background

*Notation*. Union and intersection of sets $\mathcal {A}_1$ and $\mathcal {A}_2$ are denoted by $\mathcal {A}_1 \cup \mathcal {A}_2$ and $\mathcal {A}_1 \cap \mathcal {A}_2$, respectively. The empty set is denoted by $\emptyset$. Set $\mathbb {R}$ is the space of real numbers, $\mathbb {R}_{\ge }$ the space of nonnegative real numbers, and $\mathbb {R}_{>}$ the space of positive real numbers. Similarly, ${\mathbb {Z}}$ is the space of integer numbers, ${\mathbb {Z}}_{\ge }$ the space of nonnegative integer numbers, and ${\mathbb {Z}}_{>}$ the space of positive integer numbers. When convenient, Euclidean vector $x : \{1, 2, \ldots , N\} \rightarrow \mathbb {R}$ is displayed in the column-form $\textbf{x} = (x_1, x_2, \ldots , x_N)^{\top } \in \mathbb {R}^{N} = \mathbb {R}^{N \times 1}$, where $x_i = x(i)$ and $\cdot ^{\top }$ denotes the transpose operator; the zero vector is given by $\textbf{0} = (0, 0, \ldots , 0)^{\top } \in \mathbb {R}^N$. Given any two sequences $p, q : {\mathbb {Z}}_{\ge }^N \rightarrow \mathbb {R}$, we define a bilinear form $\langle p, q \rangle \equiv \sum _{\textbf{x} \in {\mathbb {Z}}_{\ge }^N} p(\textbf{x}) q(\textbf{x})$; the $l_1$-norm of *p* is given by $\Vert p \Vert _{l_1} = \sum _{\textbf{x} \in {\mathbb {Z}}_{\ge }^N} |p(\textbf{x})|$. Kronecker-delta function centered at $x_0 \in {\mathbb {Z}}$, denoted by $\delta _{\cdot ,x_0} : {\mathbb {Z}} \rightarrow [0,1]$, is defined by $\delta _{x,x_0} = 1$ if $x = x_0$, and $\delta _{x,x_0} = 0$ if $x \ne x_0$. Given two functions $f, g : \mathbb {R}_{>} \rightarrow \mathbb {R}$, we write $f(\varepsilon ) = o(g(\varepsilon ))$ as $\varepsilon \rightarrow 0$ if $\lim _{\varepsilon \rightarrow 0} f(\varepsilon )/g(\varepsilon ) = 0$; we write $f(\varepsilon ) = O(g(\varepsilon ))$ as $\varepsilon \rightarrow 0$ if $\lim _{\varepsilon \rightarrow 0} |f(\varepsilon )|/|g(\varepsilon )| < \infty$.

### Biochemical reaction networks

We consider reaction networks $\mathcal {R} = \mathcal {R}(\mathcal {X})$ firing in well-mixed unit-volume reactors under mass-action kinetics (Feinberg 1979), involving *N* biochemical species $\mathcal {X} = \{X_1, X_2, \ldots , X_N\}$ interacting via *M* reactions, given by

69 $$\begin{aligned} \mathcal {R}(\mathcal {X})&= \bigcup _{j = 1}^M \left( \sum _{i = 1}^{N} \nu _{j,i} X_i \xrightarrow []{ k_j} \sum _{i = 1}^N \bar{\nu }_{j,i} X_i \right) . \end{aligned}$$

Here, $k_j \in \mathbb {R}_{>}$ is the *rate coefficient* of the *j*-reaction, and we let $\textbf{k} \equiv (k_1, k_2, \ldots , k_M)^{\top } \in \mathbb {R}_{>}^M$. Integers $\nu _{j, l}, \bar{\nu }_{j, l} \in {\mathbb {Z}}_{\ge }$ are the *reactant* and *product stoichiometric coefficients* of the species $X_l$ in the *j*-reaction, respectively, and we let $\varvec{\nu }_j \equiv (\nu _{j,1}, \nu _{j,2}, \ldots , \nu _{j,N})^{\top } \in {\mathbb {Z}}_{\ge }^N$ and $\bar{\varvec{\nu }}_j \equiv (\bar{\nu }_{j,1}, \bar{\nu }_{j,2}, \ldots , \bar{\nu }_{j,N})^{\top } \in {\mathbb {Z}}_{\ge }^N$. If $\varvec{\nu }_j = \textbf{0}$ (respectively, $\bar{\varvec{\nu }}_j = 0$), then the reactant (respectively, product) of the *j*-reaction is the zero species, denoted by $\varnothing$, representing species that are not explicitly modelled or non-biochemical processes. When convenient, we denote two irreversible reactions $(\sum _{l = 1}^N \nu _{i, l} X_l \xrightarrow []{k_{i}} \sum _{l = 1}^N \bar{\nu }_{i, l} X_l) \in \mathcal {R}$ and $(\sum _{l = 1}^N \bar{\nu }_{i, l} X_l \xrightarrow []{k_{j}} \sum _{l = 1}^N \nu _{i, l} X_l) \in \mathcal {R}$ jointly as the single reversible reaction . The *order of j-reaction* from network $\mathcal {R}$ is given by $\langle \textbf{1}, \varvec{\nu }_{j} \rangle \in {\mathbb {Z}}_{\ge }$. The *order of reaction network* $\mathcal {R}$ is given by the order of its highest-order reaction; $\mathcal {R}$ of order higher than two is said to be a *higher-order* network.

### Stochastic model of reaction networks

Under suitable conditions, copy-numbers of the biochemical species from (69), denoted by $\textbf{X}(t) = (X_1(t), X_2(t),\ldots , X_N(t))^{\top } \in {\mathbb {Z}}_{\ge }^{N}$, where $t \in \mathbb {R}_{\ge }$ is the time-variable, can be modelled as a continuous-time discrete-space Markov chain (Gillespie 1992). The probability-mass function (PMF) $p(\textbf{x},t)$, i.e. the probability that the copy-number vector $\textbf{X}(t) \in {\mathbb {Z}}_{\ge }^N$ at time $t > 0$ is given by $\textbf{x} \in {\mathbb {Z}}_{\ge }^N$, satisfies a partial difference-differential equation, called the *chemical master equation* (CME) (Erban et al. 2019; Van Kampen 2007), given by

70 $$\begin{aligned} \frac{\textrm{d}}{\textrm{d} t} p(\textbf{x},t) = \mathcal {L} p(\textbf{x},t)&= \sum _{j = 1}^M (E_{\textbf{x}}^{-\Delta \mathbf {\textbf{x}}_j} - 1) \big (\alpha _j(\textbf{x}) p(\textbf{x},t) \big ). \end{aligned}$$

Here, the *step operator* $E_{\textbf{x}}^{\Delta \mathbf {\textbf{x}}} = \prod _{i = 1}^N E_{x_i}^{\Delta x_i}$ is such that $E_{\textbf{x}}^{\Delta \mathbf {\textbf{x}}} f(\textbf{x}) = f(\textbf{x} + \Delta \textbf{x})$ for every sequence $f : {\mathbb {Z}}^N \rightarrow \mathbb {R}$, where $\Delta \textbf{x}_j = (\varvec{\bar{\nu }}_j - \varvec{\nu }_j) \in {\mathbb {Z}}^N$ is the *reaction vector* of the *j*-reaction. Function $\alpha _j : {\mathbb {Z}}_{\ge }^N \rightarrow \mathbb {R}_{\ge }$ is the *propensity function* of the *j*-reaction, and reads

71 $$\begin{aligned} \alpha _{j}(\textbf{x})&= k_{j} \textbf{x}^{\underline{\varvec{\nu }_j}} = k_j \prod _{i = 1}^N x_i^{\underline{\nu _{j,i}}}, \end{aligned}$$

where $x_i^{\underline{\nu _{j,i}}} = x_i (x_i - 1) (x_i - 2) \ldots (x_i - \nu _{j i} + 1)$, with the convention that $x_i^{\underline{0}} \equiv 1$ for all $x_i \in {\mathbb {Z}}_{\ge }$.

Linear operator $\mathcal {L} : \mathcal {D}(\mathcal {L}) \subseteq l_1 \rightarrow l_1$, defined in (70), is called the *forward operator*, where $l_1 = \{p : {\mathbb {Z}}_{\ge }^N \rightarrow \mathbb {R} \, | \, \sum _{\textbf{x} \in {\mathbb {Z}}_{\ge }^N} |p(\textbf{x})| < \infty \}$ and $\mathcal {D}(\mathcal {L})$ is a suitable linear subspace of $l_1$. Null-space of $\mathcal {L}$ is denoted by $\mathcal {N}(\mathcal {L}) = \{p : {\mathbb {Z}}_{\ge }^N \rightarrow \mathbb {R} \, | \, \mathcal {L} p = 0\}$. The adjoint (Kreyszig 1989) of $\mathcal {L}$, denoted by $\mathcal {L}^* : \mathcal {D}(\mathcal {L}^*) \subseteq l_{\infty } \rightarrow l_{\infty }$, where $l_{\infty } = \{q : {\mathbb {Z}}_{\ge }^N \rightarrow \mathbb {R} \, | \, \sup _{\textbf{x} \in {\mathbb {Z}}_{\ge }^N} |q(\textbf{x})| < \infty \}$ and $\sup _{\textbf{x} \in {\mathbb {Z}}_{\ge }^N}$ denotes the supremum over ${\mathbb {Z}}_{\ge }^N$, is called the *backward operator* and reads

72 $$\begin{aligned} \mathcal {L}^{*} q(\textbf{x})&= \sum _{j = 1}^M \alpha _j(\textbf{x}) (E_{\textbf{x}}^{+\Delta \mathbf {\textbf{x}}_j} - 1) q(\textbf{x}). \end{aligned}$$

## Formal perturbation analysis of network (39)–(40)

Let $\textbf{x} = (x_1, x_2, \ldots , x_m, x_{m+1}, \ldots , x_N)^{\top } \in {\mathbb {Z}}_{\ge }^{N}$ be the vector of copy-numbers for the species $\mathcal {X} = \{X_1, X_2, \ldots , X_m, X_{m+1}, \ldots , X_N \}$, and $\textbf{y} = (y_1, y_2, \ldots , y_{n-2})^{\top } \in {\mathbb {Z}}_{\ge }^{n-2}$ the copy-number vector for the auxiliary species $\mathcal {Y} = \{Y_1, Y_2, \ldots , Y_{n-2}\}$ from the network (39)–(40). Let us introduce new variables $\bar{\textbf{x}} = (\bar{x}_1, \bar{x}_2, \ldots , \bar{x}_m, x_{m+1}, \ldots , x_N)^{\top } \in {\mathbb {Z}}_{\ge }^{N}$ as follows: if there is only one distinct reactant species in the target (first) reaction from the input network (38), then

73 $$\begin{aligned} \bar{x}_1&= x_1 + \sum _{i = 1}^{n-2} (i+1) y_i, \; \; \; \text {if } m = 1. \end{aligned}$$

On the other hand, if $m \ge 2$, then

74 $$\begin{aligned} \bar{x}_l&= {\left\{ \begin{array}{ll} x_1 + \sum _{i=1}^{\nu _{1,1}} i y_{i} + \sum _{i=\nu _{1,1} + 1}^{n-2} \nu _{1,1} y_i, &{} \text {if } l = 1, \\ x_2 + \sum _{i=1}^{\nu _{1,1}} y_{i} + \sum _{i=\nu _{1,1} + 1}^{\nu _{1,1} + \nu _{1,2} - 1} (i - \nu _{1,1} + 1) y_i + \sum _{i = \nu _{1,1} + \nu _{1,2}}^{n-2} \nu _{1,2} y_i, &{} \text {if } l = 2, \text { and } m \ne 2, \\ x_l + \sum _{i= \sum _{j=1}^{l-1} \nu _{1,j}}^{-1 + \sum _{j=1}^{l} \nu _{1,j}} (1 + i - \sum _{j=1}^{l-1} \nu _{1,j}) y_i + \sum _{i= \sum _{j=1}^{l} \nu _{1,j}}^{n-2} \nu _{1,l} y_i, &{} \text {if } l \in \{3, 4, \ldots , m-1 \}, \\ x_m + \delta _{m,2} \sum _{i=1}^{\nu _{1,1}} y_{i} + \sum _{i= \delta _{m,2} + \sum _{j=1}^{m-1} \nu _{1,j}}^{n - 2} (1 + i - \sum _{j=1}^{m-1} \nu _{1,j}) y_i, &{} \text {if } l = m. \end{array}\right. } \end{aligned}$$

The CME for the output network (39)–(40), rescaled in time according to $t = \tau /\varepsilon ^{n-2}$, and expressed in terms of the new variables $\bar{\textbf{x}}$, is given by

75 $$\begin{aligned} \frac{\textrm{d}}{\textrm{d} \tau } p_{\varepsilon }(\bar{\textbf{x}},\textbf{y},\tau )&= \left( \frac{1}{\varepsilon ^{n-1}}\mathcal {L}_0 + \frac{1}{\varepsilon ^{n-2}} \sum _{i = 1}^{n-1} \mathcal {L}_{i} + \bar{\mathcal {L}}_{\rho } \right) p_{\varepsilon }(\bar{\textbf{x}},\textbf{y},\tau ). \end{aligned}$$

Here, operator $\mathcal {L}_0$ is induced by the backward reactions from $\{\mathcal {R}_i^{\varepsilon }\}_{i = 1}^{n-2}$, and reads

76 $$\begin{aligned} \mathcal {L}_0&= \sum _{i = 1}^{n-2} \mathcal {L}_0^{i}, \; \; \text {where } \mathcal {L}_0^i = \left( E_{y_{i-1}}^{-1} E_{y_{i}}^{+1} - 1 \right) y_i, \; \; \text {for all } i \in \{1, 2, \ldots , n-2 \}, \end{aligned}$$

where we define $y_0 \equiv 1$, and identify $E_{y_{0}}^{\pm 1}$ with the identity operator; $\sum _{i = 1}^{n-2} \mathcal {L}_{i}$ and $\mathcal {L}_{n-1}$ are induced by the forward reactions from $\{\mathcal {R}_i^{\varepsilon }\}_{i = 1}^{n-2}$ and the reaction $\mathcal {R}_{n-1}$, respectively, and read

77 $$\begin{aligned} \mathcal {L}_i&= {\left\{ \begin{array}{ll} \left( E_{y_{i-1}}^{+1} E_{y_{i}}^{-1} - 1 \right) y_{i-1} \alpha _i(\bar{\textbf{x}},\textbf{y}), &{} \text {if } i \in \{1, 2, \ldots , n-2 \}, \\ \left( E_{\bar{\textbf{x}}}^{-\Delta \bar{\textbf{x}}} E_{\textbf{y}}^{-\Delta \textbf{y}} - 1 \right) y_{n - 2} \alpha _{n - 1}(\bar{\textbf{x}}, \textbf{y}), &{} \text {if } i = (n-1), \end{array}\right. } \end{aligned}$$

where $\Delta \textbf{y} = (\Delta y_1, \Delta y_2, \ldots , \Delta y_{n-3}, \Delta y_{n-2})^{\top } = (\tilde{\gamma }_1, \tilde{\gamma }_2, \ldots , \tilde{\gamma }_{n-3}, \tilde{\gamma }_{n-2} - 1)^{\top }$, $\Delta \bar{\textbf{x}}$ is obtained by applying the difference operator $\Delta$ on (73)–(74), and $\{y_{i-1} \alpha _i(\bar{\textbf{x}},\textbf{y}) \}_{i = 1}^{n-1}$ are the propensity functions expressed in terms of the new variables $\bar{\textbf{x}}$; finally, operator $\bar{\mathcal {L}}_{\rho }$ is induced by the residual network $\mathcal {R}_{\rho }$ with suitably rescaled rate coefficients:

78 $$\begin{aligned} \bar{\mathcal {L}}_{\rho }&= \sum _{j = 2}^M \left( E_{\bar{\textbf{x}}}^{- \Delta \textbf{x}_j} - 1 \right) \frac{1}{\varepsilon ^{n-2}} \beta _j(\bar{\textbf{x}},\textbf{y}), \end{aligned}$$

where $\Delta \textbf{x}_j = (\bar{\varvec{\nu }}_j - \varvec{\nu }_j)$, and $\{\beta _j(\bar{\textbf{x}},\textbf{y})\}_{j = 2}^M$ are the propensity functions of the residual network expressed via $\bar{\textbf{x}}$. Let us note that only $\mathcal {L}_{n-1}$ and $\bar{\mathcal {L}}_{\rho }$ act on the variable $\bar{\textbf{x}}$, while the rest of the operators act only on $\textbf{y}$.

Let us write the solution of (75) as a perturbation series

79 $$\begin{aligned} p_{\varepsilon }(\bar{\textbf{x}},\textbf{y},\tau ) = \sum _{i = 0}^{n-1} \varepsilon ^i p_i(\bar{\textbf{x}},\textbf{y},\tau ) + \ldots . \end{aligned}$$

Substituting (79) into (75), and equating terms of equal powers in $\varepsilon$, and defining $p_{-1}(\bar{\textbf{x}},\textbf{y},\tau ) \equiv 0$, one obtains the following system of *n* equations:

80 $$\begin{aligned} \mathcal {O} \left( \frac{1}{\varepsilon ^{n-j}} \right) : \; \mathcal {L}_{0} p_{j-1}(\bar{\textbf{x}},\textbf{y},\tau )&= - \sum _{i = 1}^{n-1} \mathcal {L}_{i} p_{j-2}(\bar{\textbf{x}},\textbf{y},\tau ), \, \, \, \, \text {for all } j \in \{1, 2, \ldots , n-1 \}, \nonumber \\ \mathcal {O}(1): \; \mathcal {L}_{0} p_{n-1}(\bar{\textbf{x}},\textbf{y},\tau )&= \left( \frac{\textrm{d}}{\textrm{d} \tau } - \bar{\mathcal {L}}_{\rho } \right) p_{0}(\bar{\textbf{x}},\textbf{y},\tau ) - \sum _{i = 1}^{n-1} \mathcal {L}_{i} p_{n-2}(\bar{\textbf{x}},\textbf{y},\tau ). \end{aligned}$$

*Order* $1/\varepsilon ^{n-1}$ *equation*. Operator $\mathcal {L}_0$, given in (76), acts and depends only on the variable $\textbf{y}$, and each summand $\mathcal {L}_0^{i}$ is multiplied on the right by a factor $y_i$; therefore

81 $$\begin{aligned} p_0(\bar{\textbf{x}},\textbf{y},\tau )&= p_0(\bar{\textbf{x}},\tau ) \prod _{i=1}^{n-2} \delta _{y_i,0}, \; \; \text {where } \sum _{\bar{\textbf{x}}} p_0(\bar{\textbf{x}},\tau ) = 1, \; \; \text {for all } \tau \ge 0. \end{aligned}$$

*Order* $1/\varepsilon ^{n-2}$ *equation*. Substituting (81) into the right-hand side, one obtains

82 $$\begin{aligned} \left( \sum _{i = 1}^{n-1} \mathcal {L}_{i} \right) p_0(\bar{\textbf{x}},\textbf{y},\tau )&= \mathcal {L}_{1} p_0(\bar{\textbf{x}},\textbf{y},\tau ) = p_0(\bar{\textbf{x}},\tau ) \left( \mathcal {L}_1 \delta _{y_1,0} \right) \prod _{i=2}^{n-2} \delta _{y_i,0}, \end{aligned}$$

where we use the fact that each of the operators $\{\mathcal {L}_{i}\}_{i = 2}^{n-1}$ from (77) is multiplied on the right by a nonconstant factor $y_{i-1}$, and we use the identity $y_i \delta _{y_i,0} \equiv 0$.

Let us write the solution of the $\mathcal {O}(1/\varepsilon ^{n-2})$ equation from (80) in the separable form

83 $$\begin{aligned} p_1(\bar{\textbf{x}},\textbf{y},\tau )&= p_0(\bar{\textbf{x}},\tau ) p_1(y_1; \, \bar{\textbf{x}}) \prod _{i=2}^{n-2} \delta _{y_i, 0}, \end{aligned}$$

so that

84 $$\begin{aligned} \mathcal {L}_0 p_1(\bar{\textbf{x}},\textbf{y},\tau )&= \mathcal {L}_0^1 p_1(\bar{\textbf{x}},\textbf{y},\tau ) = p_0(\bar{\textbf{x}},\tau ) \prod _{i=2}^{n-2} \delta _{y_i, 0} \mathcal {L}_0^1 p_1(y_1; \, \bar{\textbf{x}}). \end{aligned}$$

Substituting (82) and (84) into the $\mathcal {O}(1/\varepsilon ^{n-2})$ equation, and using the operator equality $(E_{y_1}^{-1} - 1) = - (E_{y_1}^{+1} - 1) E_{y_1}^{-1}$, one obtains

85 $$\begin{aligned} \left( \prod _{i=2}^{n-2} \delta _{y_i, 0} \right) (E_{y_1}^{+1} - 1) \left( y_1 p_1(y_1; \, \bar{\textbf{x}}) - E_{y_1}^{-1} \alpha _1(\bar{\textbf{x}},\textbf{y}) \delta _{y_1, 0} \right)&= 0. \end{aligned}$$

Equation (85) is identically satisfied if $(y_2, y_3, \ldots , y_{n-2}) \ne \textbf{0}_{n-3}$, where $\textbf{0}_{n}$ is the zero element of ${\mathbb {Z}}_{\ge }^{n}$. On the other hand, if $(y_2, y_3, \ldots , y_{n-2}) = \textbf{0}_{n-3}$, the general solution satisfies

86 $$\begin{aligned} y_1 p_1(y_1; \, \bar{\textbf{x}})&= E_{y_1}^{-1} \alpha _1 \left( \bar{\textbf{x}},(y_1, \textbf{0}_{n-3}) \right) \delta _{y_1, 0} = \alpha _1 \left( \bar{\textbf{x}}, \textbf{0} \right) \delta _{y_1, 1}, \end{aligned}$$

and is given by

87 $$\begin{aligned} p_1(y_1; \, \bar{\textbf{x}})&= c_1(\bar{\textbf{x}}) \delta _{y_1,0} + \alpha _1(\bar{\textbf{x}}, \textbf{0}) \delta _{y_1,1}, \; \; \text {for all } c_1 : {\mathbb {Z}}_{\ge } \rightarrow \mathbb {R}. \end{aligned}$$

*Order* $1/\varepsilon ^{n-3}$ *equation*. It follows from (77) and (83) that

88 $$\begin{aligned} \left( \sum _{i = 1}^{n-1} \mathcal {L}_{i} \right) p_1(\bar{\textbf{x}},\textbf{y},\tau )&= \left( \mathcal {L}_{1} + \mathcal {L}_{2} \right) p_1(\bar{\textbf{x}},\textbf{y},\tau ) = p_0(\bar{\textbf{x}},t) \Big ( (\mathcal {L}_1 + \mathcal {L}_2) p_1(y_1; \, \bar{\textbf{x}}) \delta _{y_2, 0} \Big ) \prod _{i=3}^{n-2} \delta _{y_i, 0}. \end{aligned}$$

Let us write the solution of the $\mathcal {O}(1/\varepsilon ^{n-3})$ equation from (80) in the separable form

89 $$\begin{aligned} p_2(\bar{\textbf{x}},\textbf{y},\tau )&= p_0(\bar{\textbf{x}},\tau ) p_2(y_1, y_2; \, \bar{\textbf{x}}) \prod _{i=3}^{n-2} \delta _{y_i, 0}, \end{aligned}$$

so that

90 $$\begin{aligned} \mathcal {L}_0 p_2(\bar{\textbf{x}},\textbf{y},\tau )&= \left( \mathcal {L}_0^1 + \mathcal {L}_0^2 \right) p_2(\bar{\textbf{x}},\textbf{y},\tau ) = p_0(\bar{\textbf{x}},\tau ) \prod _{i=3}^{n-2} \delta _{y_i, 0} \left( \mathcal {L}_0^1 + \mathcal {L}_0^2 \right) p_2(y_1, y_2; \, \bar{\textbf{x}}). \end{aligned}$$

Substituting (88) and (90) into the $\mathcal {O}(1/\varepsilon ^{n-3})$ equation, and using the operator equalities $(E_{y_1}^{-1} - 1) = - (E_{y_1}^{+1} - 1) E_{y_1}^{-1}$ and $(E_{y_1}^{+1} E_{y_2}^{-1} - 1) = - (E_{y_1}^{-1} E_{y_2}^{+1} - 1) E_{y_1}^{+1} E_{y_2}^{-1}$, one obtains

91 $$\begin{aligned} 0&= \left( \prod _{i=3}^{n-2} \delta _{y_i, 0} \right) (E_{y_1}^{+1} - 1) \left[ y_1 p_2(y_1, y_2; \, \bar{\textbf{x}}) - E_{y_1}^{-1} \alpha _1(\bar{\textbf{x}},\textbf{y}) \delta _{y_2, 0} p_1(y_1; \, \bar{\textbf{x}}) \right] \nonumber \\&+ \left( \prod _{i=3}^{n-2} \delta _{y_i, 0} \right) (E_{y_1}^{-1} E_{y_2}^{+1} - 1) \left[ y_2 p_2(y_1, y_2; \, \bar{\textbf{x}}) - E_{y_1}^{+1} E_{y_2}^{-1} \alpha _2(\bar{\textbf{x}},\textbf{y}) \delta _{y_2, 0} y_1 p_1(y_1; \, \bar{\textbf{x}}) \right] . \end{aligned}$$

Equation (91) is identically satisfied if $(y_3, y_4, \ldots , y_{n-2}) \ne \textbf{0}_{n-4}$. On the other hand, if $(y_3, y_4, \ldots , y_{n-2}) = \textbf{0}_{n-4}$, the general solution satisfies

92 $$\begin{aligned} y_1 p_2(y_1, y_2; \, \bar{\textbf{x}})&= E_{y_1}^{-1} \alpha _1 \left( \bar{\textbf{x}},(y_1,y_2,\textbf{0}_{n-4}) \right) \delta _{y_2, 0} p_1(y_1; \, \bar{\textbf{x}}) \nonumber \\&= \left( E_{y_1}^{-1} \alpha _1 \left( \bar{\textbf{x}},(y_1, \textbf{0}_{n-3}) \right) \right) \left( c_1(\bar{\textbf{x}}) \delta _{y_1,1} + \alpha _1(\bar{\textbf{x}}, \textbf{0}) \delta _{y_1,2} \right) \delta _{y_2, 0}, \end{aligned}$$

and

93 $$\begin{aligned} y_2 p_2(y_1, y_2; \, \bar{\textbf{x}})&= E_{y_1}^{+1} E_{y_2}^{-1} \alpha _2 \left( \bar{\textbf{x}},(y_1,y_2,\textbf{0}_{n-4}) \right) \delta _{y_2, 0} \left( y_1 p_1(y_1; \, \bar{\textbf{x}}) \right) \nonumber \\&= E_{y_1}^{+1} E_{y_2}^{-1} \alpha _2 \left( \bar{\textbf{x}},(y_1,y_2,\textbf{0}_{n-4}) \right) \delta _{y_2, 0} \left( E_{y_1}^{-1} \alpha _1 \left( \bar{\textbf{x}},(y_1, \textbf{0}_{n-3}) \right) \delta _{y_1, 0} \right) \nonumber \\&= \alpha _1 \left( \bar{\textbf{x}}, \textbf{0} \right) \alpha _2 \left( \bar{\textbf{x}},(1, \textbf{0}_{n-3}) \right) \delta _{y_1, 0} \delta _{y_2, 1}, \end{aligned}$$

where we use (87) when going from the first to the second line in (92), and (86) when going from the first to the second line in (93). Hence, solutions to (92)–(93) are given by

94 $$\begin{aligned} p_2(y_1, y_2; \, \bar{\textbf{x}})&= c_2(\bar{\textbf{x}}) \delta _{y_1,0} \delta _{y_2,0} + c_1(\bar{\textbf{x}}) \alpha _1(\bar{\textbf{x}}, \textbf{0}) \delta _{y_1,1} \delta _{y_2,0} + \frac{1}{2} \alpha _1(\bar{\textbf{x}}, \textbf{0}) \alpha _1(\bar{\textbf{x}}, (1, \textbf{0}_{n-3})) \delta _{y_1,2} \delta _{y_2,0} \nonumber \\&+ \alpha _1(\bar{\textbf{x}}, \textbf{0}) \alpha _2(\bar{\textbf{x}}, (1, \textbf{0}_{n-3})) \delta _{y_1,0} \delta _{y_2,1}, \; \; \text {for all } c_2 : {\mathbb {Z}}_{\ge } \rightarrow \mathbb {R}. \end{aligned}$$

Since the right-hand side of (94) is a linear combination of a particular solution of the inhomogeneous linear equation (91) and the general solution of the underlying homogeneous equation, it follows that (94) is the general solution of (91).

*Order* $1/\varepsilon ^{n-i}$ *equation*, $i \in \{4, \ldots , n-1 \}$. One can inductively proceed to the higher-order equations from (80), with the solutions of the $\mathcal {O}(1/\varepsilon ^{n-i})$ equation written in the separable form

95 $$\begin{aligned} p_{i-1}(\bar{\textbf{x}},\textbf{y},\tau )&= p_0(\bar{\textbf{x}},\tau ) p_{i-1}(y_1, \ldots , y_{i-1}; \, \bar{\textbf{x}}) \prod _{j=i}^{n-2} \delta _{y_j, 0}, \; \; \; \text {for all } i \in \{1, 2, \ldots , n-1 \}, \end{aligned}$$

with the convention that $\prod _{i = a}^{b} f(i) = 1$ if $a > b$, where *f* is an arbitrary function, and $p_{0}(y_{0}; \, \bar{\textbf{x}}) \equiv 1$ (see also equations (81), (83) and (89)). The results (86) and (93) generalize to

96 $$\begin{aligned} y_{i} p_{i}(y_1, \ldots , y_i; \, \bar{\textbf{x}})&= \left( \prod _{j = 1}^{i-1} \delta _{y_{j}, 0} E_{y_{j-1}}^{+1} \alpha _j \Big (\bar{\textbf{x}}, (y_1, \ldots , y_j, \textbf{0}) \Big ) \right) E_{y_{i-1}}^{+1} E_{y_{i}}^{-1} \alpha _{i} \Big (\bar{\textbf{x}}, (y_1, \ldots , y_i, \textbf{0}) \Big ) \delta _{y_{i}, 0}, \end{aligned}$$

for all $i \in \{1, 2, \ldots , n-2 \}$, where we have simplified the notation via $\alpha _j(\bar{\textbf{x}}, (y_1, \ldots , y_j, \textbf{0})) = \alpha _j(\bar{\textbf{x}}, (y_1, \ldots , y_j, \textbf{0}_{n-2-j}))$. In particular, taking $i = (n-2)$ in (96), one obtains

97 $$\begin{aligned} y_{n-2} p_{n-2}(y_1, \ldots , y_{n-2}; \, \bar{\textbf{x}})&= \left( \prod _{j = 1}^{n-2} \alpha _j \left( \bar{\textbf{x}}, (\textbf{0}_{j-2}, 1, \textbf{0}_{n-(j+1)})\right) \right) \left( \prod _{j= 1}^{n-3} \delta _{y_j,0} \right) \delta _{y_{n-2},1}, \end{aligned}$$

with the convention that $\alpha _1 (\bar{\textbf{x}}, (\textbf{0}_{-1}, 1, \textbf{0}_{n -2})) \equiv \alpha _1 (\bar{\textbf{x}}, \textbf{0}_{n -2})$ and $\alpha _2 (\bar{\textbf{x}}, (\textbf{0}_{0}, 1, \textbf{0}_{n -3}))$ $\equiv \alpha _2 (\bar{\textbf{x}}, (1,\textbf{0}_{n -3}))$. In words, propensity function $\alpha _j$ is evaluated at $y_{j-1} = 1$, and $y_i = 0$ for $i \ne (j-1)$ in (97). As we shortly show, the effective CME depends on $p_{n-2}$ only via the product $y_{n-2} p_{n-2}$; see equation (100).

*Order* 1 *equation*. Analogous to Sect. 2.1, each equation from (80) is a finite-dimensional system of linear equations, so that the Fredholm alternative theorem holds. Let $\mathcal {L}_0^*$ denote the adjoint operator corresponding to $\mathcal {L}_0$, and let $\mathcal {L}_i^*$ denote the adjoint operator corresponding to $\mathcal {L}_i$; it follows from (72), (76) and (77) that $\mathcal {N}(\mathcal {L}_0^*) = \{1\}$ and that $1 \in \mathcal {N}(\mathcal {L}_i^*)$ for all $i \in \{1, 2, \ldots , n-2\}$. Hence, applying $\sum _{\textbf{y}} = \langle 1, \cdot \rangle _{\textbf{y}}$ on the $\mathcal {O}(1)$ equation from (80), one obtains the solvability condition

98 $$\begin{aligned} \frac{\textrm{d}}{\textrm{d} \tau } p_{0}(\bar{\textbf{x}},\tau )&= \left\langle 1, \mathcal {L}_{n-1} p_{n-2}(\bar{\textbf{x}},\cdot ,\tau ) \right\rangle _{\textbf{y}} + \left\langle 1, \bar{\mathcal {L}}_{\rho } p_{0}(\bar{\textbf{x}},\cdot ,\tau ) \right\rangle _{\textbf{y}}. \end{aligned}$$

Using (78) and (81), the second term on the right-hand side from (98) becomes

99 $$\begin{aligned} \left\langle 1, \bar{\mathcal {L}}_{\rho } p_{0}(\bar{\textbf{x}},\cdot ,\tau ) \right\rangle _{\textbf{y}}&= \sum _{j = 2}^M \left( E_{\bar{\textbf{x}}}^{- \Delta \textbf{x}_j} - 1 \right) \frac{1}{\varepsilon ^{n-2}} p_{0}(\bar{\textbf{x}},\tau ) \left\langle \prod _{i=1}^{n-2} \delta _{y_i,0}, \beta _j(\bar{\textbf{x}},\textbf{y})\right\rangle _{\textbf{y}} \nonumber \\&= \sum _{j = 2}^M \left( E_{\bar{\textbf{x}}}^{- \Delta \textbf{x}_j} - 1 \right) \frac{1}{\varepsilon ^{n-2}} \beta _j(\bar{\textbf{x}},0) p_{0}(\bar{\textbf{x}},\tau ). \end{aligned}$$

On the other hand, using (77) and (97), the first term on the right-hand side from (98) becomes

100 $$\begin{aligned} \left\langle 1, \mathcal {L}_{n-1} p_{n-2}(\bar{\textbf{x}},\cdot ,\tau ) \right\rangle _{\textbf{y}}&= \left( E_{\bar{\textbf{x}}}^{-\Delta \bar{\textbf{x}}} - 1 \right) p_{0}(\bar{\textbf{x}},\tau ) \Big \langle \alpha _{n-1}(\bar{\textbf{x}}, \textbf{y}), y_{n-2} p_{n-2}(\textbf{y}; \, \bar{\textbf{x}}) \Big \rangle _{\textbf{y}}, \nonumber \\&= \prod _{j = 1}^{n-1} \alpha _j \left( \bar{\textbf{x}}, (\textbf{0}_{j-2}, 1, \textbf{0}_{n-(j+1)})\right) , \end{aligned}$$

where $$\alpha _{n-1} (\bar{\textbf{x}}, (\textbf{0}_{n-3}, 1, \textbf{0}_{0})) \equiv \alpha _{n-1} (\bar{\textbf{x}}, (\textbf{0}_{n-3}, 1))$$.

If $m = 1$, using (73), it follows that $\alpha _1(\bar{x}_1, \textbf{y}) = \kappa _1 (\bar{x}_1 - \sum _{i = 1}^{n-2} (i+1) y_i) (\bar{x}_1 - \sum _{i = 1}^{n-2} (i+1) y_i - 1)$; hence, $\alpha _1(\bar{x}_1, \textbf{0}_{n -2}) = \kappa _1 \bar{x}_1 (\bar{x}_1 - 1)$. Similarly, $\alpha _j \left( \bar{x}_1, (\textbf{0}_{j-2}, 1, \textbf{0}_{n - (j+1)})\right) = \kappa _j (\bar{x}_1 - j)$ for all $j \in \{2, 3, \ldots , n-1 \}$. Hence, in this case,

101 $$\begin{aligned} \prod _{j = 1}^{n-1} \alpha _j \left( \bar{\textbf{x}}, (\textbf{0}_{j-2}, 1, \textbf{0}_{n - (j+1)})\right)&= \left( \prod _{j = 1}^{n-1} \kappa _j \right) \bar{x}_1^{\underline{n}}, \; \; \; \text {if } m = 1. \end{aligned}$$

Similarly, in the case $m \ge 2$, using (74), one obtains

102 $$\begin{aligned} \prod _{j = 1}^{n-1} \alpha _j \left( \bar{\textbf{x}}, (\textbf{0}_{j-2}, 1, \textbf{0}_{n - (j+1)})\right)&= \left( \prod _{j = 1}^{n-1} \kappa _j \right) \prod _{l = 1}^m \bar{x}_l^{\underline{\nu _l}}, \; \; \; \text {if } m \ge 2. \end{aligned}$$

Substituting (99)–(102) into (98), and changing the time back to the original scale, $\tau = \varepsilon ^{n-2} t$, one obtains the effective CME

103 $$\begin{aligned} \frac{\textrm{d}}{\textrm{d} t} p_0(\bar{\textbf{x}},t)&= \left( \left( E_{\bar{\textbf{x}}}^{-\Delta \bar{\textbf{x}}} - 1 \right) \left( \varepsilon ^{n-2} \prod _{j = 1}^{n-1} \kappa _j \right) \prod _{l = 1}^m \bar{x}_l^{\underline{\nu _l}} + \mathcal {L}_{\rho }\right) p_0(\bar{\textbf{x}},t), \end{aligned}$$

where $\mathcal {L}_{\rho }$ is induced by the residual network $\mathcal {R}_{\rho }$.

### Kinetic and stoichiometric conditions

In order for the effective CME (103) to match the CME of the input network (38), the kinetic condition (41) must hold. Furthermore, we require that $\Delta \bar{\textbf{x}} = \Delta \textbf{x}_1 = (\varvec{\bar{\nu }_1} - \varvec{\nu }_1)$. In the special case when $(\tilde{\gamma }_1, \tilde{\gamma }_2, \ldots , \tilde{\gamma }_{n-3}) = \textbf{0}$, applying the difference operator $\Delta$ on (74), one obtains

104 $$\begin{aligned} \Delta \bar{x}_l&= \Delta x_l + (\nu _{1,l} - \delta _{l,m}) \Delta y_{n-2} \nonumber \\&= (\tilde{\nu }_l -\delta _{l,m}) + (\nu _{1,l} - \delta _{l,m}) (\tilde{\gamma }_{n-2} - 1), \; \; \; \text {for all } l \in \{1, 2, \ldots , m\}; \end{aligned}$$

imposing the matching condition $\Delta \bar{x}_l = (\bar{\nu }_{1,l} - \nu _{1,l})$, one obtains the stoichiometric conditions (42).

## Proof of Theorem 4.1

Consider the output network (39)–(40) under the scaling (52), with the CME given by

105 $$\begin{aligned} \frac{\textrm{d}}{\textrm{d} t} p_{\varepsilon }(\bar{\textbf{x}},\textbf{y},t)&= \mathcal {L}_{\varepsilon } p_{\varepsilon }(\bar{\textbf{x}},\textbf{y},t) = \left( \frac{1}{\varepsilon } \mathcal {L}_0 + \frac{1}{\varepsilon ^{\frac{n-2}{n-1}}} \sum _{i = 1}^{n-1} \mathcal {L}_{i} + \mathcal {L}_{\rho } \right) p_{\varepsilon }(\bar{\textbf{x}},\textbf{y},t), \end{aligned}$$

where $\mathcal {L}_{\rho }$ is induced by $\mathcal {R}_{\rho }$. Substituting into (105) the perturbation series

106 $$\begin{aligned} p_{\varepsilon }(\bar{\textbf{x}},\textbf{y},t) = \sum _{i = 0}^{n-1} \varepsilon ^{\frac{i}{n-1}} p_i(\bar{\textbf{x}},\textbf{y},t) + \ldots , \end{aligned}$$

one obtains

107 $$\begin{aligned} \mathcal {O} \left( \frac{1}{\varepsilon ^{1 - \frac{i}{n-1}}} \right) : \; \mathcal {L}_{0} p_{i}(\bar{\textbf{x}},\textbf{y},t)&= - \left( \sum _{i = 1}^{n-1} \mathcal {L}_{i}\right) p_{i-1}(\bar{\textbf{x}},\textbf{y},t), \, \, \, \, \text {for all } i \in \{0, 1, \ldots , n-2 \}, \nonumber \\ \mathcal {O}(1): \; \mathcal {L}_{0} p_{n-1}(\bar{\textbf{x}},\textbf{y},t)&= \left( \frac{\textrm{d}}{\textrm{d} t} - \mathcal {L}_{\rho } \right) p_{0}(\bar{\textbf{x}},\textbf{y},t) - \left( \sum _{i = 1}^{n-1} \mathcal {L}_{i}\right) p_{n-2}(\bar{\textbf{x}},\textbf{y},t). \end{aligned}$$

Having the same form, systems (107) and (80) have solutions of the same form; in particular

108 $$\begin{aligned} p_0(\bar{\textbf{x}},\textbf{y},t)&= p_0(\bar{\textbf{x}},t) \delta _{\textbf{y},\textbf{0}}, \end{aligned}$$

with

109 $$\begin{aligned} \frac{\textrm{d}}{\textrm{d} t} p_0(\bar{\textbf{x}},t)&= \left( \left( E_{\bar{\textbf{x}}}^{-\Delta \bar{\textbf{x}}} - 1 \right) \left( \prod _{j = 1}^{n-1} \bar{\kappa }_j \right) \prod _{l = 1}^m \bar{x}_l^{\underline{\nu _l}} + \mathcal {L}_{\rho } \right) p_0(\bar{\textbf{x}},t), \end{aligned}$$

where $\Delta \bar{\textbf{x}}$ is obtained by applying the difference operator $\Delta$ on (73)–(74); see e.g. (104).

### Proof

For every bounded set $\mathbb {S}_{\bar{x}} \times \mathbb {S}_{y} \subset {\mathbb {Z}}_{\ge }^{N + (n-2)}$, $p_{\varepsilon }(t) = p_{\varepsilon }(\cdot ,\cdot ,t)$ is a finite-dimensional vector. By choosing $\mathbb {S}_y \supseteq [0,C]^{n-2}$ with a sufficiently large $C > 0$, one can reverse the arguments from Sect. 2 and recover the same finite-dimensional vectors $\{p_i(t) = p_i(\cdot ,\cdot ,t)\}_{i = 1}^{n-1}$ such that system (107) is satisfied; in what follows, we also let $p_0(t) = p_0(\cdot ,\cdot ,t)$. Let us define a remainder function $r_{\varepsilon }(t) = r_{\varepsilon }(\cdot ,\cdot ,t)$ via

110 $$\begin{aligned} p_{\varepsilon }(t)&= \sum _{i = 0}^{n-1} \varepsilon ^{\frac{i}{n-1}} p_i(t) + r_{\varepsilon }(t). \end{aligned}$$

Substituting (110) into (105), and using (107), together with $p_{\varepsilon }(0) = p_0(0)$, one obtains an initial-value problem for the remainder:

111 $$\begin{aligned} \frac{\textrm{d}}{\textrm{d} t} r_{\varepsilon } (t) - \mathcal {L}_{\varepsilon } r_{\varepsilon } (t)&= \sum _{i = 1}^{n-1} \varepsilon ^{\frac{i}{n-1}} f_i(t), \; \; \; r_{\varepsilon }(0) = - \sum _{i = 1}^{n-1} \varepsilon ^{\frac{i}{n-1}} p_i(0), \end{aligned}$$

where

112 $$\begin{aligned} f_1(t)&= \mathcal {L}_{\rho } p_1(t) -\frac{\textrm{d}}{\textrm{d} t} p_1(t) + \sum _{i = 1}^{n-1} \mathcal {L}_{i} p_{n-1}(t), \; \; \; f_i(t) =\mathcal {L}_{\rho } p_i(t) - \frac{\textrm{d}}{\textrm{d} t} p_i(t), \; \; \text {for all } i \in \{2, 3, \ldots , n-1\}. \end{aligned}$$

Solving (111), applying the $l^1$-norm on $\mathbb {S}_{\bar{x}} \times \mathbb {S}_{y} \subset {\mathbb {Z}}_{\ge }^{N + (n-2)}$, the triangle inequality, and using the fact that $\Vert e^{\mathcal {L}_{\varepsilon }^n t} \Vert _{l_1(\mathbb {S}_{\bar{x}} \times \mathbb {S}_{y})} \le 1$, one obtains

113 $$\begin{aligned} \Vert r_{n} (t) \Vert _{l_1(\mathbb {S}_{\bar{x}} \times \mathbb {S}_{y})}&\le \sum _{i = 1}^{n-1} \varepsilon ^{\frac{i}{n-1}} \left( \Vert p_i(0)\Vert _{l_1(\mathbb {S}_{\bar{x}} \times \mathbb {S}_{y})} + t \, \underset{0 \le s \le t}{\text {max}} \left\| f_i(s) \right\| _{l_1(\mathbb {S}_{\bar{x}} \times \mathbb {S}_{y})}\right) . \end{aligned}$$

The PMF $p_0(t; \, \varvec{\bar{\kappa }}, \textbf{k}_{\rho })$ and its time-derivatives are bounded for all $(\varvec{\bar{\kappa }}, \textbf{k}_{\rho }) \in {\mathbb {K}}$ and $t \in [0,T]$, since the PMF satisfies (109) on $S_{\bar{x}} \subset {\mathbb {Z}}_{\ge }^N$. Hence, $\Vert r_{\varepsilon } (t; \, \varvec{\bar{\kappa }}, \textbf{k}_{\rho }) \Vert _{l_1(\mathbb {S}_{\bar{x}} \times \mathbb {S}_{y})} = \mathcal {O}(\varepsilon ^{1/(n-1)})$ as $\varepsilon \rightarrow 0$ for all $(\varvec{\bar{\kappa }}, \textbf{k}_{\rho }) \in {\mathbb {K}}$ and $t \in [0,T]$ which, together with (110), implies the joint-PMF error bound $\left\| p_{\varepsilon }(\cdot ,\cdot ,t; \, \, \varvec{\bar{\kappa }}, \textbf{k}_{\rho }) - p_0(\cdot ,\cdot ,t; \, \varvec{\bar{\kappa }}, \textbf{k}_{\rho }) \right\| _{l_1(\mathbb {S}_{\bar{x}} \times \mathbb {S}_{y})}$ $\le c \, \varepsilon ^{1/(n-1)}$. Statement of Theorem 4.1 then follows by using the fact that the CME (109) is identical to the CME of the input network (38) when the conditions (41) and (42) are satisfied, and by using $p_0(\bar{\textbf{x}},\textbf{y},t) = p_0(\textbf{x},\textbf{y},t)$.
